# Operationalizing an open-source dashboard for communicating results of wastewater-based surveillance

**DOI:** 10.1016/j.mex.2023.102299

**Published:** 2023-07-27

**Authors:** Dustin Hill, Christopher Dunham, David A. Larsen, Mary Collins

**Affiliations:** a Department of Public Health, Syracuse University, Syracuse, NY, USA; b School of Information Studies, Syracuse University, Syracuse, NY, USA; c School of Marine and Atmospheric Sciences, Sustainability Studies Division, Stony Brook University, Stony Brook, NY, USA; d Institute for Advanced Computational Science, Stony Brook University, Stony Brook, NY, USA

**Keywords:** *An open-source development guide for web-based data dashboards for public health*, COVID-19, Public communication, Data sharing, Dashboard, R Shiny

## Abstract

COVID-19 saw the expansion of public health tools to manage the pandemic. One tool that saw extensive use was the public health dashboard, web-based visualization tools that communicate information to users in easy-to-read graphics. Dashboards were widely used prior to the pandemic, but COVID-19 saw expanded use and development. To date, dashboards have become an important part of public health surveillance programs around the world helping decisionmakers use data to evaluate different public health metrics including caseloads, hospitalizations, and environmental surveillance results from testing wastewater. Wastewater surveillance provides community-based, spatially relevant data on disease trends within communities to assess the scale of infection in a region, which makes it an excellent candidate for dashboard development to improve public health. We developed a dashboard for New York State's wastewater surveillance program using open-source, reproducible web programming. The dashboard we developed has been used for the COVID-19 response in New York, and our methods can be adapted to other programs and pathogens. We provide:

• descriptions of how the dashboard was developed and maintained
• specific guidance for reproducing our dashboard in other areas and for other pathogens
• fully reproducible code with step-by-step instructions for researchers and professionals to make their own data dashboards

## Graphical abstract

**Figure:**
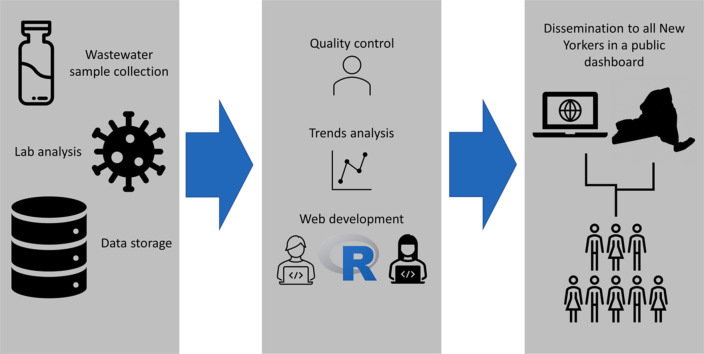

Specifications table

**Table:** 

Subject area:	Environmental Science
More specific subject area:	*Public health communication tools*
Name of your method:	*An open-source development guide for web-based data dashboards for public health*
Name and reference of original method:	Chang W, Borges Ribeiro B. shinydashboard: Create Dashboards with “shiny.” 2021.
Resource availability:	*R statistical software*https://www.r-project.org/ *R shiny*https://www.rstudio.com/products/shiny/ *R studio*https://posit.co/download/rstudio-desktop/ *R code tutorial (GitHub page)*https://github.com/dthill196/SARS-2-Dashboard-Tutorial *R code tutorial (webpage)*https://dthill196.github.io/SARS-2-Dashboard-Tutorial/ *New York State Dashboard*https://mbcolli.shinyapps.io/SARS2EWSP/#

**Method details**

## Introduction and context

Surveillance of infectious diseases provides information on the current burden of disease, trends in transmission, and can help identify outbreaks [1]. During the COVID-19 pandemic, wastewater-based infectious disease surveillance (wastewater surveillance) gained increasing popularity [2], [3], [4]. Data collected through wastewater surveillance can be presented in different ways to communicate risk to institutional and public decisionmakers, but there is no clear consensus on the most effective visualization methods [5]. In emergent infectious disease situations, real-time data collection and reporting requires using straightforward visualizations that communicate risk quickly in a way relevant to decisionmakers and the public [5]. Online dashboards are convenient as a tool to show these visuals, and the COVID-19 pandemic has seen their widespread deployment.

Pairing wastewater surveillance with dashboards can provide decisionmakers and the public with timely data reporting that are not biased by inaccurate case counts — a key advantage of wastewater surveillance [[6], [7]]. While numerous wastewater surveillance data dashboards have been created around the world and supervised by different organizations, such as local governments [8] and universities [9], most lack reproducibility and the flexibility to be applied to other pathogens. In our view, this is a missed opportunity because these tools should not remain in use only during present crises but should be flexible to enable application to potential new threats. In addition, with the transition away from the COVID-19 pandemic emergency response, wastewater surveillance is well suited toward informing upon resurgence of disease and widespread monitoring of new variants of concern.

We use New York State's surveillance of SARS-CoV-2, the virus that causes COVID-19, as our example pathogen for demonstrating the development process and methods for creating a dashboard for wastewater data. We discuss the metrics developed that communicate the state of transmission in different sampling locations including current alert levels, trends in the data indicating where transmission might be going, and geographic locations for all sampling points. We conclude with a discussion of the New York State dashboard's strengths and limitations as well as future directions for our tool and how it can be adapted to address other public health concerns.

## Methods

### Selection of software and web services

We considered a variety of different dashboard development software, however, our team decided to use the R coding language [10] and R Shiny package [11] to build the final dashboard. R Shiny was selected because it is open source, easily shareable, and widely used by our team members, including the research scientists handling wastewater data. This helped streamline the translation of the methods used for analysis into visuals suitable for a dashboard. In addition, the use of R allowed for rapid dashboard development that took advantage of our team's skillset. For a full list of all R packages used and their purpose, please see Table 1.

**Table 1:** R packages used to create the dashboard.

*Group*	*Package*	*Use*
Shiny app support packages	shiny [11]	Package for building the interactive web components for the Shiny app.
	shinydashboard [12]	Provides layout for the application with a sidebar, title space, as well as making it easy to layout content in the body of the application with default features.
	shinydashboardPlus [13]	Adds functions to enhance the shinydashboard package.
	shinyBS [14]	Adds mouse-over tooltips to figures, buttons, and features in the application.
	shinyjs [15]	Allows the app to read and use JavaScript applications including the use of toggle buttons and hiding content until a button is activated.
	shinyalert [16]	Assists with html code within the application.
	shinycssloaders [17]	Adds loading icons for map and plots while the app is loading in the web browser and when generating new plots.
	htmltools [18]	Use html code within the application. Used in creation of text sections and loading images.
Data processing (spatial and nonspatial)	sf [19]	Load and manipulate spatial data.
	aws.s3 [20]	Read in data from Amazon Web Services S3 bucket.
	dplyr [21]	Manipulate data frames.
	tidyr [22]	Wrangling data into correct formats for the application.
	magrittr [23]	Adds additional functionality to the “pipe” operator and supports other packages like dplyr
	purrr [24]	Functions used to calculate rolling averages for case data.
	stringr [25]	Edit and manipulate strings in the data.
	lubridate [26]	Edit and manipulate dates to various formats.
Leaflet packages	leaflet [27]	Creation of the interactive map on the main page.
	leaflet.extras [28]	Enables leaflet to work with plug-ins.
Figure and table creation	ggplot2 [29]	Creation of trend plots for wastewater and case data plots.
	plotly [30]	Wrapper functions turn ggplots into interactive features in the dashboard.
	gt [31]	Creation of tables within the application.

### Database inflow and management

Laboratory testing results are delivered daily by email to a shared email inbox dedicated to this purpose. To support this project, we set up a dedicated “inbox” for laboratory testing results using the OneDrive service from Microsoft. Permission to update the inbox is provided to laboratory operators only. Similar to email, a script processes any new inbox files at regular intervals, including moving processed CSV files out of the inbox and into permanent storage. Email was kept in place because it provides a record of the chain of custody and redundancy.

For storage of processed laboratory testing results, we chose to use RSQLite (https://cran.r-project.org/web/packages/RSQLite/index.html), an R package that interfaces with the free and widely-used SQLite database software (https://www.sqlite.org/index.html). SQLite allows single-file storage of an entire database, which is helpful for backing up and sharing records. Backup copies of this database and CSV lab reports are stored on an Amazon Simple Storage Service (Amazon S3) “bucket.” Amazon S3 buckets are secure cloud object storage instances which can be managed programmatically using the R package aws.s3 [20]. Amazon S3 API “keys” used by aws.s3 functions are managed on the Amazon Web Services (AWS) console and can be configured for specific use cases, such as providing read-only access to a bucket or a single object within a bucket. Quality checking steps, such as ensuring the reported testing values are within an expected range, are performed at the time of processing laboratory reports for database storage. Records that do not meet quality control standards are automatically flagged for further review and kept from wider dissemination until approved.

Laboratory testing results are delivered as soon as laboratory testing is complete. Sampling occurs between once and three times per week at each site in NY. Units for the data are in gene copies per milliliter. Data are also normalized by human fecal indicators [32]. The data feeding the dashboard is updated daily, requiring a remote connection to the database. Rather than set up a fully remote database, which requires another layer of administration, we decided to leverage the Amazon S3 API and the small size of the data needed to run the dashboard. Code embedded in the dashboard to run at startup will transfer and store locally processed files in RDS format — a highly compressed file format native to R — only when updates to the database have been made. This code sends an API call to the S3 bucket, requesting a list of all objects in the bucket and the last time each was modified. The API call checks the modification time of the S3 instance of the dashboard data against the last locally stored copy of that data. If the S3 data were created more recently, an API call to transfer the S3 version to dashboard location is made and the new data is loaded into memory for the dashboard to use for that session. The effort to only transfer updated data, rather than the entire database, reduces latency for the dashboard user, as well as decreases cost by limiting the amount of data transferred by the API each month. An alternative to this process would be to transfer data at regular intervals only, but we chose continuous data upload to get as close as we could to real-time dissemination of results.

### Data preprocessing

Once data are organized and safely stored, the next step is to preprocess the data for use by the R Shiny application. We use two primary metrics in the New York State wastewater surveillance network, as described below. Any metrics of interest can be calculated. These metrics are calculated each time new data are deposited in the AWS server (AWS S3 bucket) and the application detects new data. The code uses AWS S3 API to check the modified time of the data file every time the dashboard is accessed via the web, and if the AWS data file is newer than the existing file, the dashboard downloads the latest file version. Once the trend and alert metrics are calculated and stored on the server, the dashboard can report them to the user for each location a sample was taken by linking to the correct geography.

Each wastewater sample is linked to the geographic location it was sampled from and these are mapped on the main page of the dashboard. Wastewater data are community-level samples and therefore do not reveal any information about individuals, making presentation of the spatial information related to the results appropriate without infringing on individual privacy. The spatial data we link to are the geographic coordinates of the sampling location and the sewershed from where that sample is drawn (the combined area of all sewers linked up to the plant and sampling location). The sewershed is displayed in the map with simplified geometry to increase performance and speed of the dashboard.

To provide context for the wastewater data, case and test positivity data are loaded directly into the application through an API call to New York State's COVID-19 database (https://coronavirus.health.ny.gov/covid-19-testing-tracker). Case counts from confirmed PCR tests per county are displayed alongside wastewater results for the county as well as with rolling averages for active cases and test positivity per day. These data, while limited due to underlying biases, show that trends in wastewater commonly follow the trends in case data providing a visual link for users. Currently, we have kept these data in the dashboard despite reduced quality of the data and, while case counts are less reliable, for New York, test positivity trends still closely follow wastewater trends providing utility for users comparing the two datasets.

The last set of data used in the dashboard are spatial layers for counties, sewersheds, and wastewater treatment plants. County boundaries were obtained from the U.S. Census Tiger/Line shapefiles database [33] and sewershed boundaries were drawn as part of a separate data collection effort [34]. Wastewater treatment plant coordinates were downloaded from the New York State Department of Environmental Conservation (DEC) website [35]. All data are linked to the sampling point and sewershed boundary, which allows users to focus on the data for their community.

### Public health metrics

To communicate information on where COVID-19 may be transmitting, and trends in detection, we chose to communicate two metrics: alert levels of SARS-CoV-2 detected in wastewater and trends in detection. Alert levels were based on three categories that were found in previous research to correlate highly with geocoded case counts within the sampling areas [36]. Trends were calculated using a two-week linear trend of the change in wastewater results over time inclusive of all data points for the two-week period (including outliers). With sampling ranging from once weekly to three times weekly, each site has a minimum of three data points with up to nine data points for three-times weekly samplers. By providing the quantity of wastewater detections of SARS-CoV-2 alongside the trend, users can see where a community is currently at regarding estimated levels of the virus and whether transmission is increasing or decreasing.

### R shiny code

#### Shiny dashboard

R Shiny is a package that uses R code to build interactive web applications [11]. We used the package shinydashboard [12] to provide a preset structure to the dashboard including the initial layout. The base layout of shinydashboard includes a header, sidebar, and main body that can be filled with interactive content for the users. This template reduced some of the hard coding necessary to create these additional features.

#### Leaflet

One of our main aims was to adequately communicate the spatial coverage of the surveillance network in New York to show the extent of the data, but also to support a diverse user group. For example, users come from different areas (e.g., counties, cites) and, while being able to see the entire state is important, it was also important that we support a more granular view for those looking for information of most relevance to their locations. The main page of our dashboard has a Leaflet map using the R package Leaflet [27]. Leaflet is an interactive map-making software designed for use with many coding languages and the interface is simple and easy to use by dashboard visitors. Leaflet also includes built-in features allowing users to set the zoom level, move the map view in different directions, and click map locations to get more information. The information presented on a map click can be customized in the dashboard, which allowed our team to add important information to points of interest, such as SARS-CoV-2 detection level and trend, as well as metadata about the treatment plant sampling location including estimated population served. Leaflet can work with Environmental Systems Research Institute or ESRI shapefiles as well as table data with geocoordinates. The majority of user interaction on our dashboard is via the Leaflet map, which lets users click locations of interest to then learn more about detection level and trends for that location. This gives the user the ability to navigate to anywhere in New York and increases the most relevant user population to be anyone within the state of New York.

#### Trend plots

We built trend plots using the R package ggplot2 [29] and made them interactive using the R package plotly [30]. The plotly program can be applied to static plots to make them interactive and increases their usability for interactive dashboards. By making the trend plots interactive, we let the users view all the data over time for their region and manipulate the plot to view specific time periods in which they are interested.

#### Interactivity

The dashboard's Leaflet map has many interactive elements, which lets users select points and regions of interest where wastewater sampling is occurring. When users select points on the map, counterpart trend plots are updated that correspond to the point. In addition, different trend plots are available to the user and selectable via sidebar radio buttons.

#### Code management

The R source code is a collaborative effort and is shared and managed in a GitHub repository to ensure version control. GitHub is very common code sharing management option used for version control of development-related files. It also allowed for formal code collaboration practices. The current repository is private due to data sharing restrictions, however, a duplicate repository with the raw code is publicly accessible from this link (https://dthill196.github.io/SARS-2-Dashboard-Tutorial/). The duplicate repository includes all the code used to generate the dashboard as well as supporting data.

### R shiny server

The dashboard is hosted on shinyapps.io, an app hosting service. This service comes with different payment tiers for support. We selected shinyapps.io for hosting because it gave us the greatest freedom for managing the dashboard and code allowing us to freely update the source code when necessary. In addition, shinyapps.io allows users to create as many hosted apps as desired allowing us to establish a private, staging environment app. The staging environment is accessible only to invited users and allows us to test development innovations before going live.

### Web analytics

To evaluate dashboard viewership, we ran a summary of visits using Similar Web (https://www.similarweb.com/). Similar Web is a for-profit service that summarizes website usage and provides some limited free data about website visits. We obtained a summary of site visits between the months of September 2022 and November 2022 as well as basic information about how long users stayed on the site and what links they clicked on.

## Resources and code availability

All code used to create the dashboard is publicly available at https://dthill196.github.io/SARS-2-Dashboard-Tutorial/. In addition, we created a supplemental tutorial document to explain the code and its purpose in our dashboard. The live and up-to-date dashboard is available at https://mbcolli.shinyapps.io/SARS2EWSP/# and http://www.nywastewatcher.io/.

## Dashboard implementation results and findings

**Fig. 1:**
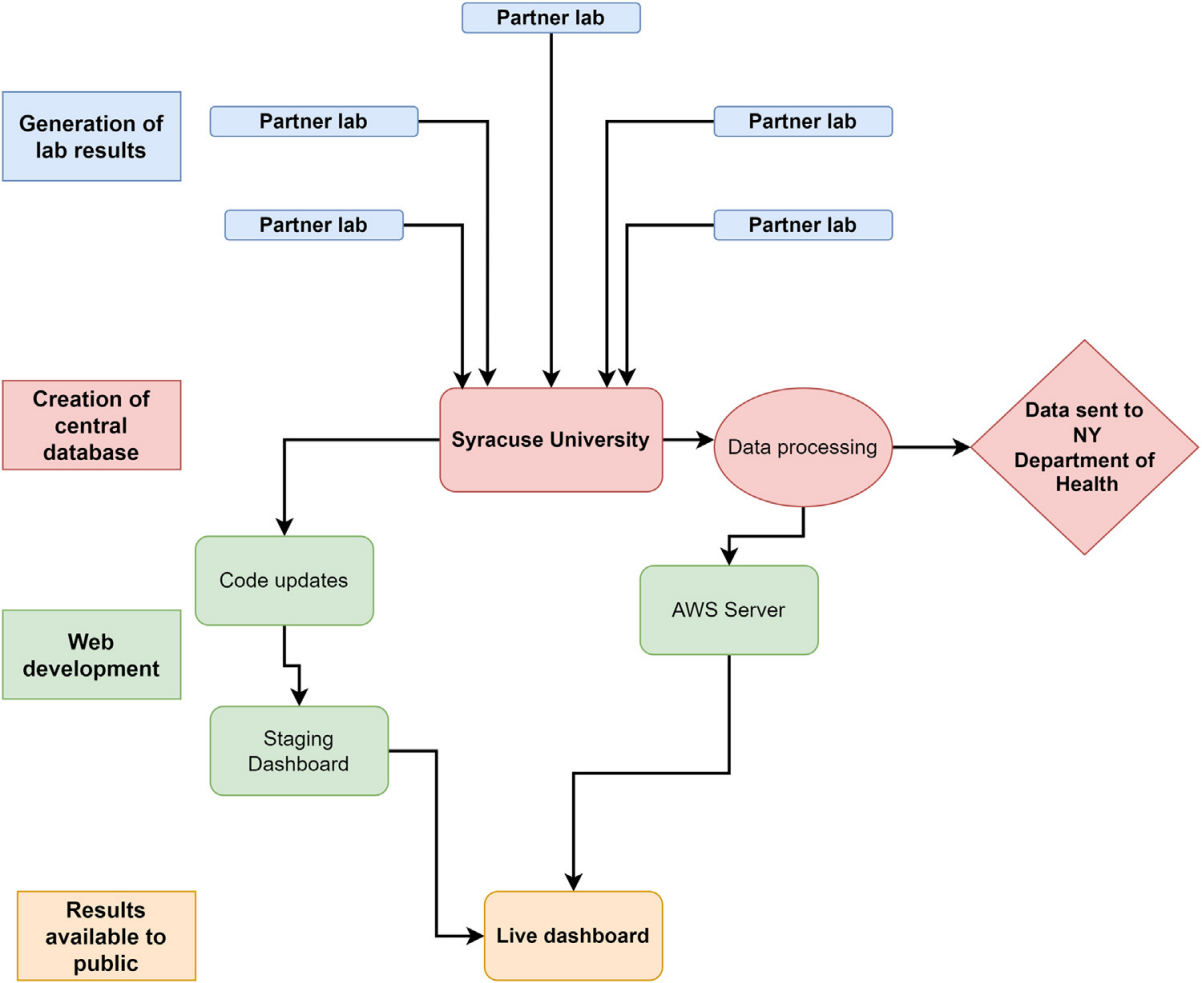
Dashboard workflow. Wastewater samples are processed by participating laboratories. Resulted data is then sent to Syracuse University for processing, management, and upload to the AWS S3 bucket before being published to the dashboard. Processed data are sent to the New York State Department of Health for submission to the National Wastewater Surveillance System [8].

We receive quantification levels for SARS-CoV-2 and associated data from the laboratories testing wastewater within 24 h of sample collection, at which point results are processed by Syracuse University, the central location for all data management (Fig. 1). The data are then delivered to the AWS S3 bucket where the dashboard code monitors for updates. Once a data update is detected, dashboard figures are updated to display the latest results. Time from sample collection to reporting to the dashboard was 60 h or less for most locations from day zero for sample collection to day three after sample collection.

**Fig. 2:**
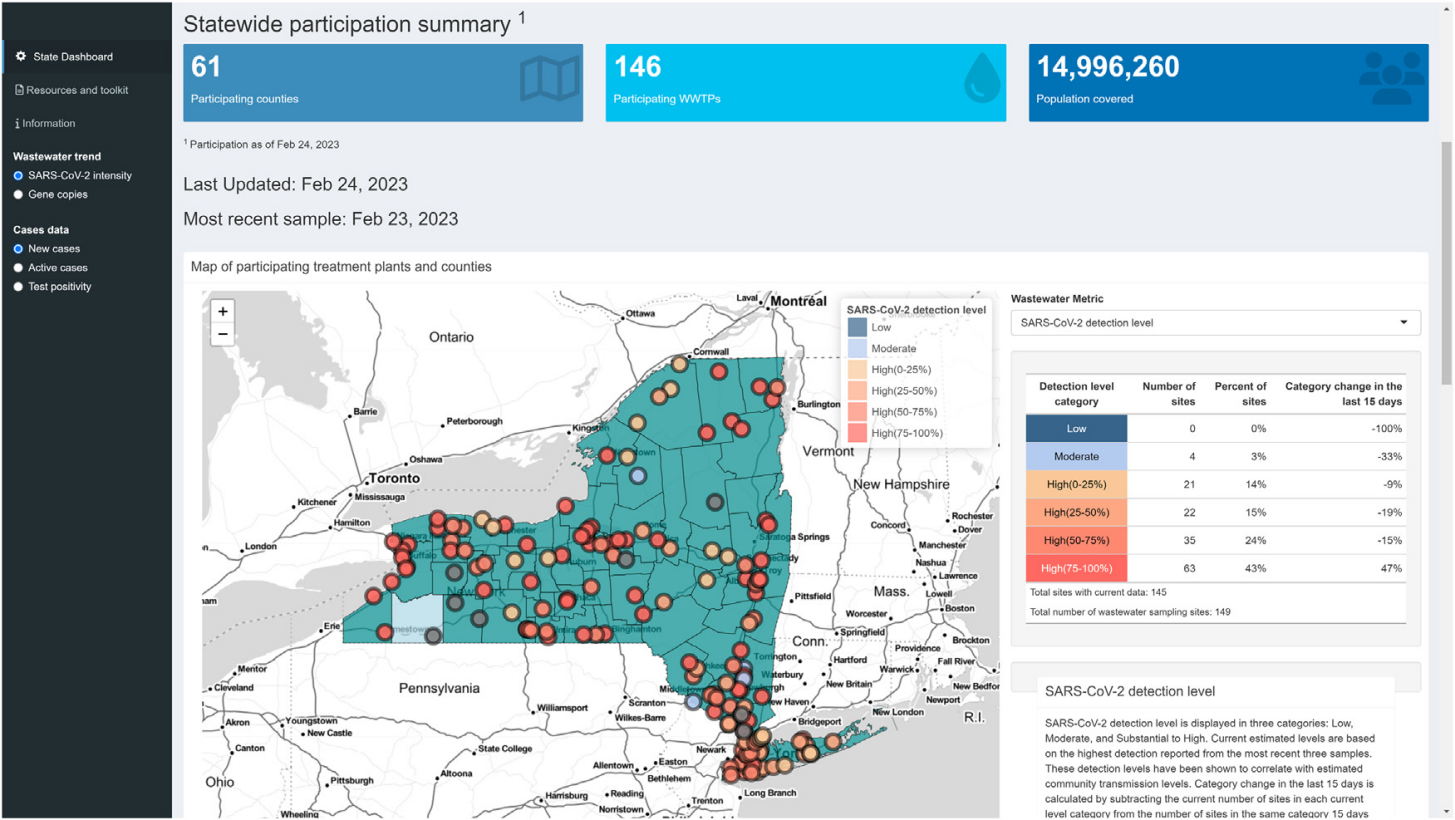
Screenshot of the dashboard landing page showing the Leaflet map on the landing page. Users can select locations by clicking around the map and zoom in to view more detail about a sampling location most relevant to their interests. The map can display different metrics, including current trend and detection levels using a dropdown on the top right.

Displayed on our Leaflet map, we link each wastewater sample to its sampling location allowing users to click on a geography of interest and show tailored results (Fig. 2). Local leadership in that jurisdiction can use these data to make public health decisions such as recommending vaccination [37] and monitor disease resurgence, and the public can use these data to guide their level of social interactions such as whether to attend large gatherings. Further, state officials use the trend information to inform regional distribution of hospital resources including treatments, preventative care messaging, and coordinate with local health departments. Further, the interactivity of the Leaflet map lets users choose where they want to examine results, increasing the relevance of the dashboard to many different users such as media outlets seeking to communicate local conditions to users like in Ithaca, NY [38]. Trends in detection were linked to each sample location and inform the relevant community about disease transmission dynamics in that area.

**Fig. 3:**
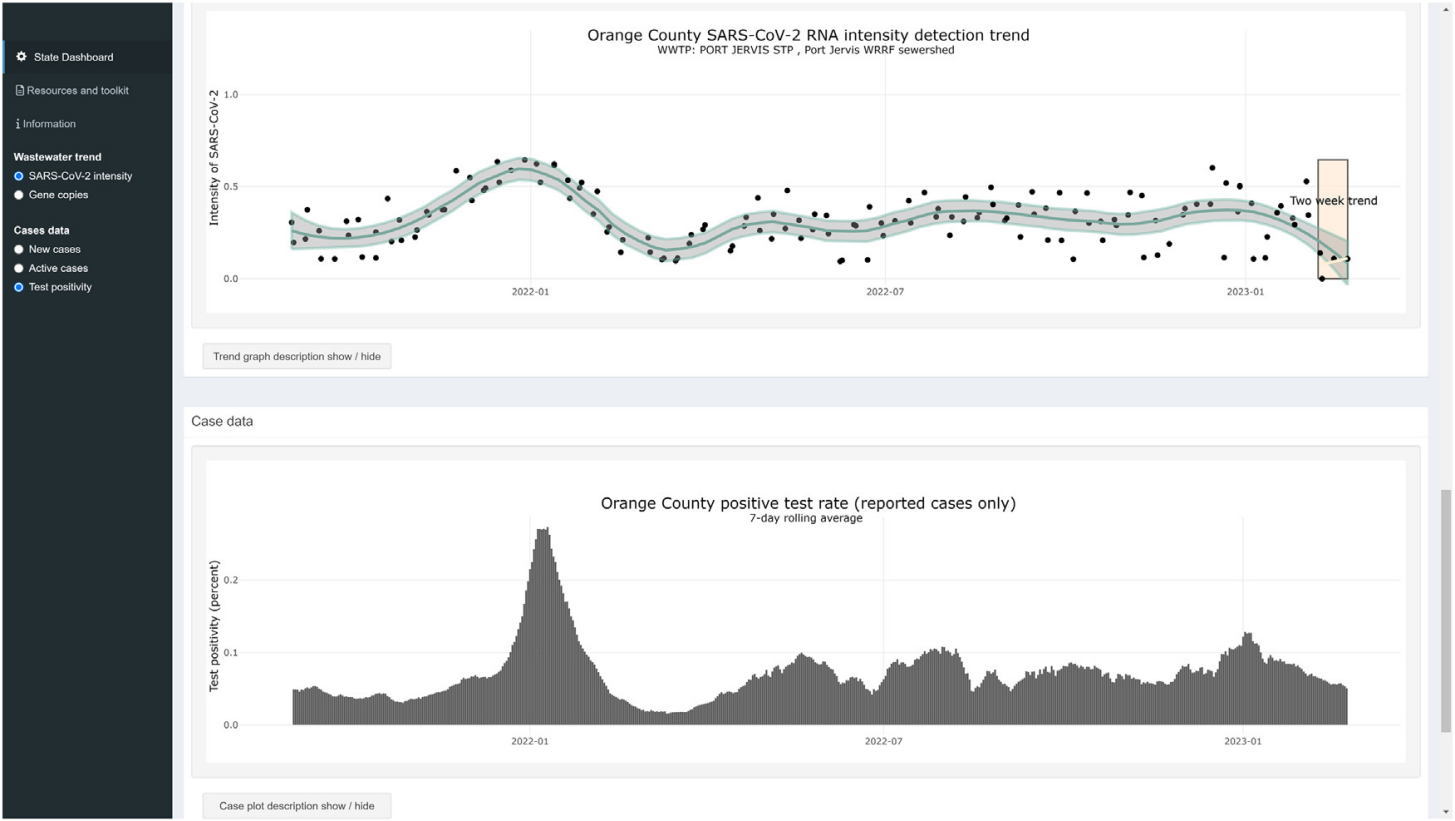
Trend in wastewater detection of SARS-CoV-2 normalized by human fecal indicator (crAssphage in this instance) to create a measure of SARS-CoV-2 “intensity” in the wastewater. Intensity follows active case trends very closely.

Detection levels are displayed to viewers in three categories: Low, Moderate, and High (high is further broken down into four quantiles for high levels determined by quantifiable detections of SARS-CoV-2). Each of these categories corresponds to CDC categories for community transmission prior to February 2022, which were low transmission (< 10 weekly cases per 100,000 population), moderate transmission (10–49 weekly cases per 100,000 population) and substantial to high transmission (> 50 weekly cases per 100,000 population). In addition, trend information is reported to the viewer as the percent change over two weeks in the normalized levels of SARS-CoV-2 in wastewater at each site. The change in the number of sites that fall within each detection level and trend category are also reported showing how transmission dynamics have changed since previous data were reported (see Fig. 2). We also display the trend in data as a plot showing the normalized amount of SARS-CoV-2 detected on each sampling day since sampling began to give viewers the opportunity to compare recent detections to past levels. Lastly, we highlight the two weeks of data used to calculate the trend information to differentiate the recent results from previous days (Fig. 3).

**Fig. 4:**
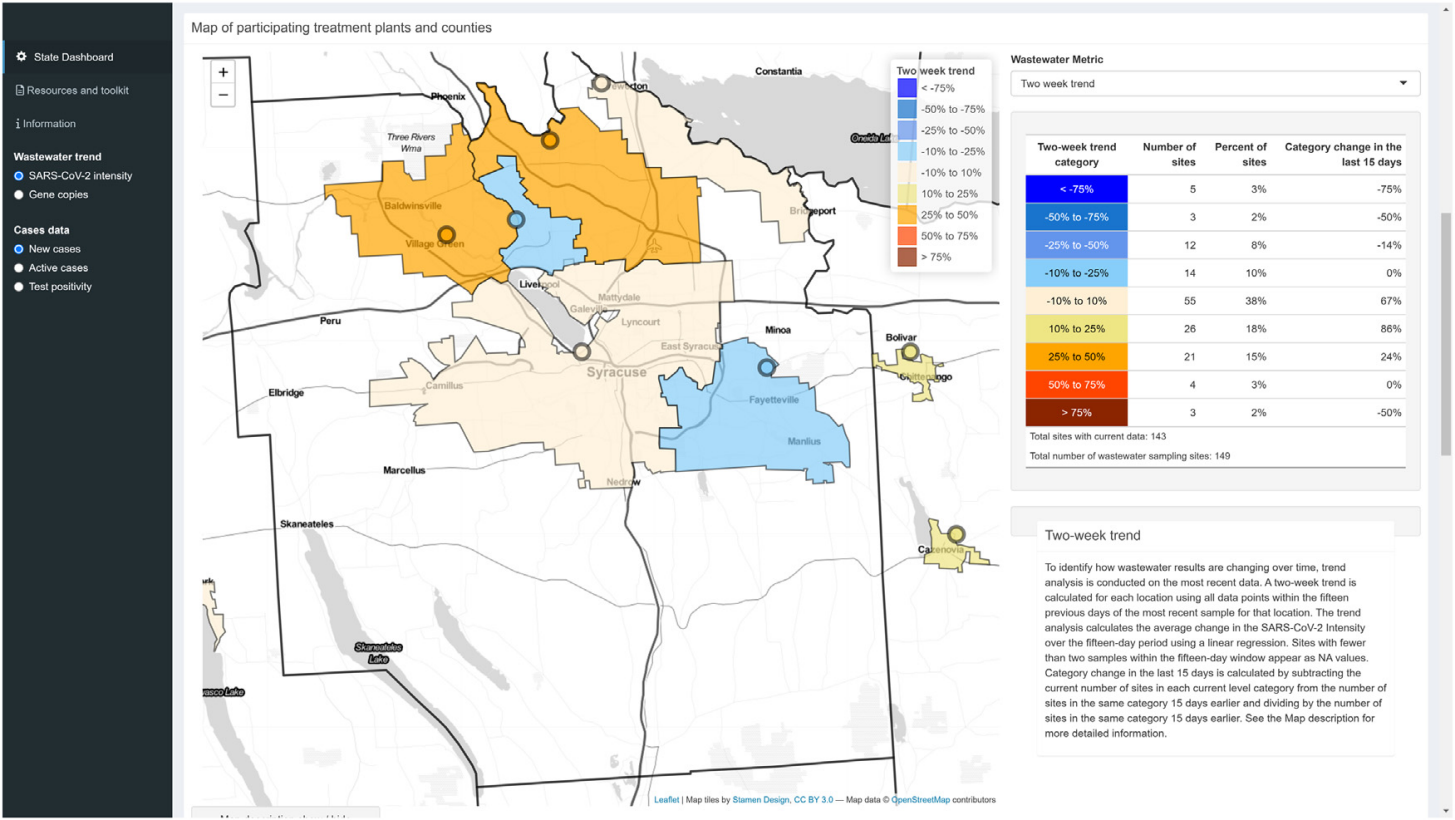
Sewershed level zoom on the Leaflet map with outlines for each treatment plant's service area and associated trend information.

All data are reported at the sampling site-level (the wastewater treatment plant) and users can “zoom in” to areas of the Leaflet map that show the borders of the treatment plant's sewershed (Fig. 4). This provides important spatial context for the users and shows what communities are contributing to the data shown.

As mentioned, we used Similar Web to assess usage of the dashboard. Between September and November 2022 our dashboard had 8373 total visits, with 62.85 percent made by mobile users and 37.15 percent by desktop users. The average duration that users spent on the website was 1 min and 49 s with 80.43 percent of users closing the website after that time. Most users (61.64 percent) visited the site through direct links, which means they may have had the website URL stored in their search engine, with 30.34 percent of users going to the site through a referral link such as from the New York State DOH website (https://www.health.ny.gov/environmental/wastewater.htm). The remaining users captured in the report linked to the site from social media (4.28 percent) or organic searches (3.73 percent). Users further engaged with links provided on the dashboard including links to New York State's health data website and the CDC's National Wastewater Surveillance website.

## Additional information

### Dashboards as public health communication tools

COVID-19 emerged just as a data revolution was occurring in public health [39]. Over the past decade, public health has increasingly provided data back to the community [[40], [41]]. Dashboards have become a preferrable way to make sense of large data that are being collected in real-time [42]. These dashboards communicate a variety of public health issues such as comparing disease burden between different geographies and reporting on differences in medical coverage between regions [43]. While dashboards have existed in the field of public health for several years, their emergence as a mainstream method to quickly communicate data came of age during the pandemic and emergency response efforts [44]. COVID-19 dashboards arose from numerous coalitions eager to support the pandemic in part due to increasingly accessible software and also because COVID-19 data were made readily available [45]. In the initial stages of the pandemic, the public largely relied on the Johns Hopkins dashboard that scraped case counts based on PCR testing from multiple government sites [46]. Over time, national and state health departments, non-governmental organizations, and even private citizens created dashboards that pulled data from multiple data streams including clinical data, such as case counts, hospitalizations, and deaths [[46], [47]], as well as data from wastewater surveillance [[2], [48]]. Some dashboards that have been developed serve the purpose of visually displaying data and trends to users without providing prescriptive action to respond to different situations [49] while others focus on forecasting changes in disease transmission [50].

At its heart, wastewater surveillance is a community-level measure. Therefore, wastewater surveillance dashboards should communicate spatially relevant data on community health dynamics [51] with wastewater surveillance results providing individuals, communities, and public health officials with important information needed to make decisions to address the spread of disease, like where to hold vaccine clinics [52], and the public may use the information to make arrangements for large gatherings or travel. Herein we presented a process for developing and maintaining a customizable dashboard through free open-source software and reproducible methods that can be applied to any pathogen or chemical surveilled in wastewater. Approaches for each target might need modifications to the method presented in order to address differences such as for chemical/drug targets that will have different data structure, sampling frequency, and intervention needs.

Public health problems such as COVID-19 require timely reporting of data, information on geographic extent of risk, and information on trends to give users the tools they need to make the best decision for their situation. Dashboards are one tool that New York State selected for collecting and disseminating wastewater surveillance data to manage the spread of COVID-19 in the state. Trend and current state of disease are two of the essential components of infectious disease surveillance that we present to users [1]. Decisionmakers rely on the improved understanding that wastewater surveillance data provide regarding the current state of the COVID-19 pandemic, including planning the locations of vaccination clinics and monitoring regional resurgence of the disease [53]. Feedback from local health departments to our development team included interest in understanding the trends presented and how they could communicate them to the public. Decisions made by public health officials in different counties across New York State that were informed by the dashboard include putting out press releases on current trends from wastewater [[37], [38]] and crafting public health messages about wastewater [54].

Using R Shiny, we were able to develop and maintain a public dashboard useable by different regions and individuals in New York State that is timely, provides information of geographic relevance, and conveys key metrics about infectious disease transmission including trends. The use of the R coding language makes the dashboard easy to update over time with the development of new analyses by the scientific team. The dashboard is flexible and versatile, and we anticipate that its infrastructure will be well suited to support of data visualization and risk communication for future public health concerns.

Based on visitor data to the website, we know that users spent an average of less than two minutes on the dashboard page. These results suggest that graphics on the dashboard must be easily interpreted, and users might be obtaining the information they need quickly. This short time span is important to keep in mind for future development to make sure new graphics are not overly complex and retain the same ease-of-use in the present dashboard. In addition, user time on the dashboard could also be linked to finding graphics confusing or being intimidated by the dashboard's design. To remedy this, a tutorial video has been added along with a PDF download for users to view and learn how to use the dashboard. In addition, we received frequent emails from users not engaged in public health practice with questions about the data (contact email is provided on the dashboard information page). This keeps our development team connected to users so that feedback can be incorporated. Further, most users arrived at the website through a direct web link (a bookmarked URL). These users were most likely public health officials or treatment plant operators that were provided the link to the dashboard as part of their onboarding procedure so they can receive continuous updates abouts about their county's surveillance status. This means that most users of the dashboard are members of the statewide network or affiliated with local health departments. Still, a little over thirty percent of users arrived at the site from a referral link from the New York DOH website, meaning that there are still new users arriving at the page. Such referral links are important for ensuring the public can find the dashboard. In addition, our low number of links from social media might suggest that more work could be done to “spread the word” about the dashboard including what and how it can be used.

It is important to note that dashboards, due to their flexibility and customizability, can literally show whatever data the creators desire. While this flexibility can be a powerful public health tool, it also means that they are potentially biased toward the skills, interests, and fields of the designers and owners [44]. As such we advocate that dashboard developers be as transparent as possible about data collection and manipulation, with open data availability being a gold-standard [[51], [55]]. In addition to designing dashboards to be useful and accurately present public health data to users, developers must consider both the resources and time needed to develop and maintain a dashboard [56] and that dashboards can outlive their usefulness [45]. Keeping these factors in mind is an important dashboard managerial effort. For example, over the course of a dashboard's useful life it will need regular maintenance, up-to-date metrics, and intervention information, and will change over time. Old and outdated information must be actively removed when no longer needed. Eventually, most dashboards will need to be properly archived and retired. Such considerations are important for ensuring optimal use of resources while maintaining relevant tools for public health action.

### Application to other pathogens and public health issues

Key steps in the process for building our dashboard to support the COVID-19 response can be applied to other public health concerns. These include data management and storage and the general workflow shown in Fig. 1. Different pathogens will have their own nuances that should be considered, and environmental toxins are quite different from infectious diseases. Expert understanding of what the wastewater surveillance data suggest is needed to develop metrics that are ready to be communicated to the public. Once metrics are developed, the data can be prepared and stored in a comparable way that we present, including the use of a readable file on a private data server that R can process and use to update the dashboard code. This is both timely and avoids an individual having to manually publish the dashboard each time new data become available. In addition, many of the features we included in our dashboard can be applied to other public health projects including the Leaflet map and trend graphs.

The Leaflet map is important in that it shows the spatial coverage of the project and gives users a clear understanding of the community linked to the sample. This can help with decision making by both public health managers and the community. Data sensitivity is an important consideration when displaying geographic data on a public dashboard. While our project uses community-level samples from wastewater, wastewater surveillance can also be applied at smaller spatial scales, including the building-level. At the level of the building, developers might consider leaving the map out of publicly released dashboard content or aggregating the data to higher levels of geography to avoid infringement on privacy. Alternatively, if geographic information is key, then developers might consider private hosting options or one internal to local health departments. Shinyapps.io does allow developers to privately deploy dashboards that are only viewable by authenticated users. This can help ensure privacy, while also letting key individuals use data to support public health policy and intervention.

The trend graphs and user interface we developed are also adaptable to other public health concerns. Substitution of figures and graphics can be done based on the project. What we have built is not prescriptive in any way, and R Shiny is flexible, allowing new users to modify and improve upon our template.

### Lessons learned and future directions

Initial prototype development of our dashboard took two months or approximately 150 person hours between two developers. The final dashboard, in its state at the time of this publication, took an additional three months and an estimated 300 person hours spread between three developers. Current maintenance is performed by one part-time data scientist that spends approximately 25 percent of their work toward dashboard maintenance (around 8 person hours per week). Another part-time data scientist works on data management to support this dashboard and other research related projects, and that involves approximately 8 person hours per week. Lastly, one supervisor contributes approximately 4 person hours per month to manage the project. In total, monthly maintenance involves approximately 68 person hours per month. Maintenance of the dashboard costs approximately $3072.67 per month ($36,872.00 per year) inclusive of personnel time ($2720.00 per month), web resources (up to $3.00 per month), and hosting on R shiny.io ($349.00 per month) (see Supplemental Table 1 for details). All development undergoes review by the project manager and a team consisting of researchers, public health officials at New York State Department of Health (NY DOH), and epidemiologists assigned to the wastewater surveillance project.

Along the way, we found testing the application before it is published to be essential. Testing the application before it was published was a key component of each stage of development. Our team did not use a staging dashboard initially, and while we were able to prepare and publish the dashboard successfully, subsequent updates did not always move seamlessly to the live dashboard. Introduction of a development step that used a staging dashboard in a private domain to test its features online became a key and valuable step to avoid live updates that might contain issues that could hamper users. Another goal we had was to directly transfer data from emails when they entered the inbox. A script could be written to automatically pull CSV attachments from emails to be processed for storage in a database as part of a future effort and this method can be used for any email service (e.g., Gmail).

Annotation of the code is easy to do in R and makes keeping a record of activity possible. Publishing the code to a GitHub repository allowed our team to collaborate and keep track of changes to maintain version control. R Shiny includes many nested functions and steps that can be difficult to disentangle if they are not clearly identified. Therefore, we took extra steps to indicate the beginning of new function calls and the closing of those same function calls in the code through annotations. This made it easier to see when something started or ended and to find errors in the code when they appeared. One other point for future development is to set up web analytics when the dashboard is made live to monitor traffic and use. While we were able to obtain some information after the dashboard was live, having built-in analytics such as through Google Analytics could have provided more immediate and long-term information on visits, usership, and public health reach. Such information is essential if you are to assess the impact of a dashboard and monitor its lifecycle to determine when a dashboard can be archived and retired.

The open-source nature of R means that our success is built on the work of others. The resulting dashboard and tutorial we have developed build upon successful code and dashboards that were developed long before COVID-19. This allowed us to focus on tailoring the dashboard to the needs of our specific project rather than having to completely start from scratch or develop our own dashboard layout. Using shinydashboard, Leaflet, and the other packages we list in Table 1 allowed us to take the essential information we wanted to communicate about SARS-CoV-2 detection and build a user interface that best communicated the data to different users.

The future of our dashboard includes further refinement of features we already have and development of new functionality. One step in the dashboard code that we would like to separate is the data preprocessing. While pre-processing data during the launch of the application does not currently hamper performance, this step could slow down the dashboard in the future with larger datasets. In addition, our dashboard has many of the components identified as standard for a dashboard on infectious diseases including providing quantitative results, but we also have areas to improve such as providing downloadable source data [[51], [55]]. The dashboard will change to reflect the most up-to-date information and tools that public health practitioners need to manage COVID-19 and understand wastewater data, including the addition of SARS-CoV-2 genetic sequencing data. While the dashboard is maintained by a university team, the funding support comes from NY DOH and all updates and changes are coordinated with state health department officials. It is the primary tool for most local health departments in NY in addition to individualized county-level reports. The reproducible nature of the dashboard that we created will make it instrumental for future pathogens and helpful for other groups looking to develop similar tools. Such tools continue to grow and evolve, offering new opportunities for public engagement, communication, and action to improve public health and community well-being.

## Ethics statements

N/A.

## CRediT authorship contribution statement

**Dustin Hill:** Conceptualization, Methodology, Software, Validation, Formal analysis, Investigation, Writing – original draft, Writing – review & editing, Visualization. **Christopher Dunham:** Methodology, Software, Validation, Resources, Data curation, Writing – review & editing. **David A. Larsen:** Conceptualization, Resources, Writing – review & editing, Supervision, Project administration, Funding acquisition. **Mary Collins:** Conceptualization, Resources, Writing – review & editing, Supervision, Project administration, Funding acquisition.

## Declaration of Competing Interest

The authors declare that they have no known competing financial interests or personal relationships that could have appeared to influence the work reported in this paper.

## Data Availability

Data will be made available on request.

## Appendix Supplementary materials

Image, application 1

Image, application 2
