# Supplementary material for: Operationalizing an open-source dashboard for communicating results of wastewater-based surveillance
Source: MethodsX. 2023 Jul 27;11:102299. doi: 10.1016/j.mex.2023.102299 (PMC10404718; doi:10.1016/j.mex.2023.102299)
Supplement: Supplementary file 1 [file mmc1.pdf]

Wastewater Surveillance Dashboard Tutorial Using R and R Shiny

Dustin T. Hill, Chris Dunham, David A. Larsen, and Mary B. Collins

2022-12-28

Contents

1 Introduction **2**

2 R packages **2**

3 Data and Preprocessing **3**

3.1 Wastewater data 3

3.2 Automatic data updates 4

3.3 Case data 5

3.4 Spatial data 7

4 User Interface **8**

4.1 Customizing Shiny Dashboard colors 10

4.2 Value boxes 11

4.3 Drop-down menu (select input) 11

5 Leaflet **12**

5.1 Leaflet interactivity 14

5.2 Leaflet proxy 14

6 Trend graphs and tables **15**

6.1 Wastewater trends 16

6.2 Case trends 18

6.3 Summary tables 18

7 Interactivity between Leaflet and plots **20**

8 Closing thoughts **21**

9 References **21**

1 Introduction

This tutorial provides the source code for the New York State Wastewater Surveillance Network's SARS-CoV-2 data dashboard. The tutorial goes through the major code chunks for processing the wastewater data, case data, and spatial data used in the R shiny application. Further, the tutorial discusses some of the techniques used to provide automatic data updates to the dashboard, how each figure was created, and how interactivity was coded into the dashboard. Portions of the source code are provided in this document. The source code is also available in an unabridged version at this GitHub page (insert link maybe). This tutorial represents the state of the dashboard on the date of publication and may not reflect the most up-to-date version of the code or dashboard. For the most up-to-date version, the authors direct you to the Git Hub page referenced above.

This tutorial has been divided into the following major sections:

1) Data and preprocessing - explanation of all data sources and storage methods, data manipulation and calculations.
2) User Interface - the source code (with annotations) for the user interface designed for the dashboard
3) Leaflet - the source code for the Leaflet map and Leaflet proxy used to display the spatial data
4) Trend graphs - the functions used to create the wastewater and case trend data
5) Interactivity between Leaflet and plots - a section with code and examples one how the interactivity was coded for between the map and trend plots
6) References and Resources - a list of references and useful resources that guided the creation of our dashboard.

2 R packages

To run the shiny app, the packages listed in Table 1 need to be installed and loaded. The shiny, shiny dashboard, and shiny dashboard plus packages are all required for creating the layout and basic interactive features. The data processing packages like dplyr and tidyr are necessary for data preprocessing when the app launches and the Leaflet packages

Table 1: R packages used to create the dashboard

Group	Package	How package is used
Shiny app support packages	shiny	Package for building the interactive web components for the shiny app.
	shiny dashboard (Chang & Borges Ribeiro, 2021)	Provides layout for the application with a sidebar, title space, as well as making it easy to layout content in the body of the application with default features.
	shinydashboardPlus (Granjon, 2021)	Adds functions to enhance the shiny dashboard package.
	shinyBS (Bailey, 2022)	Adds mouse-over tooltips to figures, buttons, and features in the application.
	shinyjs (Attali, 2021)	Allows the app to read and use JavaScript applications including the use of toggle buttons and hiding content until a button is activated.

Group	Package	How package is used
	shinyalert (Attali & Edwards, 2021)	Assists with html code within the application.
	shinycssloaders (Sail & Attali, 2020)	Adds loading icons for map and plots while the app is loading in the web browser and when generating new plots.
	htmltools (Cheng et al., 2021)	Use html code within the application. Used in creation of text sections and loading images.
Data processing (spatial and nonspatial)	sf (Pebesma, 2018)	Load and manipulate spatial data.
	aws.s3 (Leeper, 2020)	Read in data from Amazon Web Services S3 bucket.
	dplyr (Wickham et al., 2022)	Manipulate dataframes.
	tidyr (Wickham & Girlich, 2022)	Wrangling data into correct formats for the application.
	magrittr (Bache & Wickham, 2022)	Improves readability of code.
	purrr (Henry & Wickham, 2020)	Functions used to calculate rolling averages for case data.
	stringr (Wickham, 2019)	Edit and manipulate strings in the data.
	lubridate (Grolemund & Wickham, 2011)	Edit and manipulate dates to various formats.
Leaflet packages	Leaflet (Cheng et al., 2022)	Creation of the interactive map on the main page.
	Leaflet.extras (Karambelkar & Schloerke, 2018)	Enables Leaflet to work with plug-ins.
Figure and table creation	ggplot2	Creation of trend plots for wastewater and case data plots.
	plotly (Sievert, 2020)	Wrapper functions turn ggplots into interactive features in the dashboard.
	gt (Iannone et al., 2022)	Creation of tables within the application.

3 Data and Preprocessing

3.1 Wastewater data

The detection levels and two-week trend is assigned to the data as part of the preprocessing step. The methods used by the New York labs allows for classification of wastewater surveillance data for SARS-CoV-2 into three primary categories: Low, Moderate, and Substantial to High. These levels correspond to transmission levels previously described (Larsen et al. 2022). Two-week trends are calculated for each wastewater treatment plant using the following code:

```
# empty list to populate regression results within for loop
datalist2 <- list()

# loop through all current testing sites to get 2 week trends
for (i in base::unique(wastewater$SW_ID)) {

    # select sewershed
```

```
    df_reg <- wastewater %>%
        filter(SW_ID == i)

    ### two week regression

    # linear model comparing the trend value over time (intensity or raw gene
    # copies)
    model_2weeks <- lm((trend_value) ~ lubridate::as_date(Date_collected), data = df_reg)  #

    # pull out coefficients as indicators of rising or falling trend
    df_reg$TwoWeekBet <- model_2weeks$coefficients[2]

    # pull out R2 for confidence metric
    df_reg$TwoWeekR2 <- summary(model_2weeks)$r.squared

    df_reg$slope <- model_2weeks$coefficients[1]

    # add the df to the list object
    datalist2[[i]] <- df_reg
}

# convert the list to a df
wastewater.df.sewersheds <- do.call(rbind, datalist2)

# calculate genesee, allegany, and suffolk data separately
wastewater.df.sewersheds$TwoWeekBet <- wastewater.df.sewersheds$TwoWeekBet * 14  # 14 day change
```

3.2 Automatic data updates

Data are stored on an Amazon Web Services (AWS) bucket. Data are loaded directly into the app from the bucket, and we added a function that has the app scan the AWS bucket for updates. When an update is pushed to the bucket, the app then reloads the data updating all the figures and maps.

The data are read into the app using the following code:

```
check_for_new_wws_data(key = aws_key, secret = aws_key)
wastewater <- readRDS("nys.wastewater.rds")
```

The check for updates function is coded in as follows:

```
Sys.getenv('aws_bucket')
Sys.getenv('aws_key')
Sys.getenv('aws_secret')


check_for_new_wws_data = function(key, secret) {

  bucket_contents =
    get_bucket('nystatewws',
               key = key,
               secret = secret)
```

```
  wws.data.rds = bucket_contents[lapply(bucket_contents,
                                       function(x) x$Key == 'nys.wastewater.rds') %>%
                                  unlist()
                                ]

  last_update = wws.data.rds$Contents$LastModified %>%
    lubridate::as_datetime() %>%
    lubridate::with_tz(tzone = 'America/New_York')

  download_new_file = function() {
    wws.data =
      aws.s3::s3readRDS(object = 'nys.wastewater.rds',
                        bucket = 'nystatewws',
                        key = key,
                        secret = secret)
    saveRDS(wws.data, file = 'nys.wastewater.rds')
  }

  if(!file.exists('nys.wastewater.rds')) {download_new_file()}
  if(file.exists('nys.wastewater.rds') &
     last_update > lubridate::as_datetime(file.info('nys.wastewater.rds')$mtime) %>%
     lubridate::with_tz(tzone = 'America/New_York')

     ) {
    download_new_file()
  }

}
```

The check for updates function is saved as a separate R file and stored in a folder named "R" that is put in the main Git Hub repository. See Figure 1 for a screenshot of the repository layout.

NOTE: When setting up your dashboard, you will have to provide your own AWS key and secret in the appropriate code sections. You will want to store them in your environment.

3.3 Case data

Case data for New York state are downloaded through API from the state website at (DOH 2022). Three transformations are done to integrate the case data for the dashboard. First, test positivity is calculated by dividing the total number of positive tests by the total number of tests given on that day. Second, active cases are estimated by taking a rolling sum over 6 days. Third, the 7-day rolling average of active cases is calculated from the active case value. The dashboard provides users with 1) new case counts, 2) test positivity, and 3) 7-day rolling average of active cases.

```
# CASE DATA LOAD AND PREPROCESSING #

link <- paste("https://health.data.ny.gov/api/views/xdss-u53e/rows.csv?accessType=DOWNLOAD&bom=true&forr

# case data from nys website
all.cases <- read.csv(link, header = TRUE, sep = ";")
colnames(all.cases)[1] <- "test_DATE"

# data prep (date variable change and make case data numeric)
```

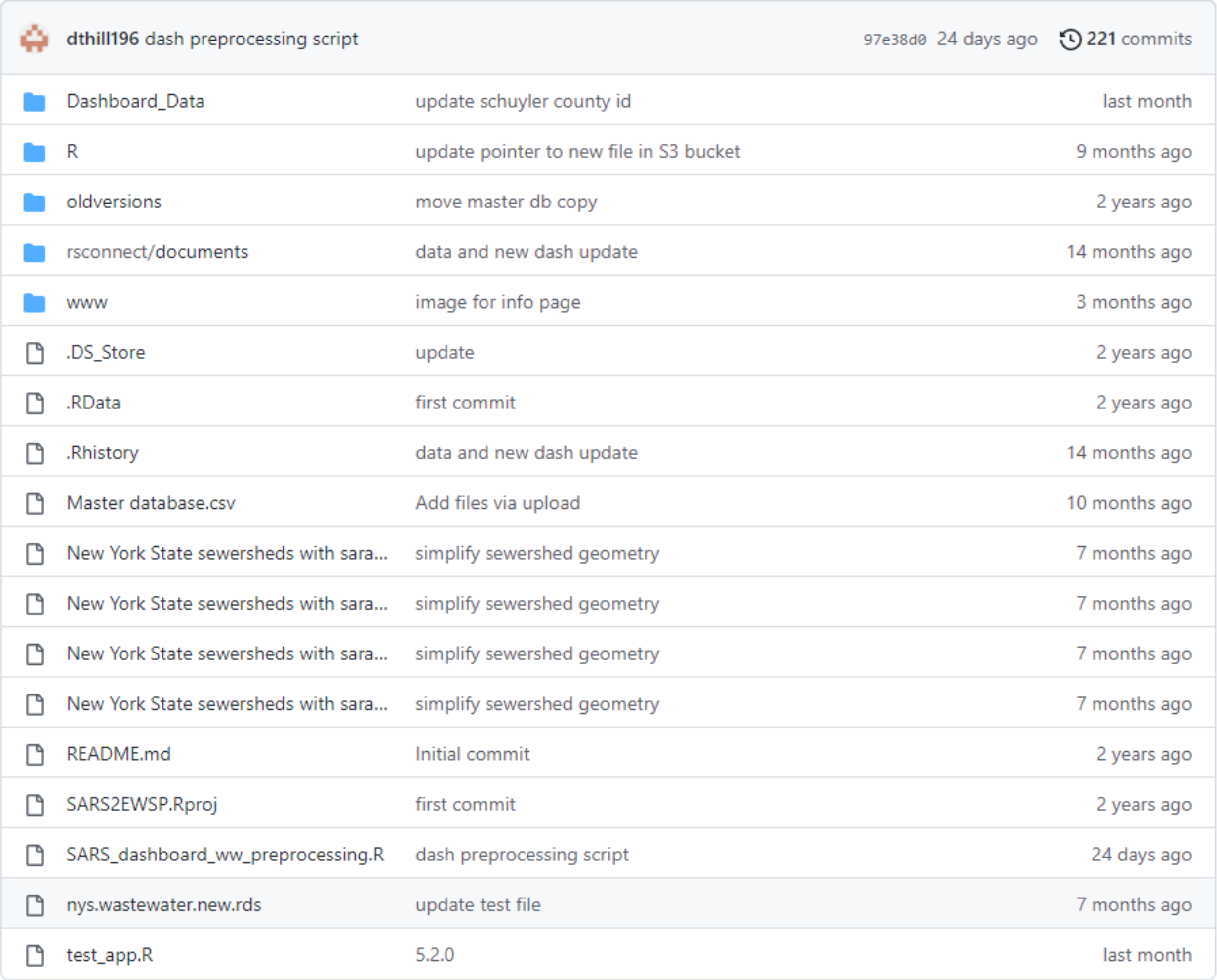

dthill196 dash preprocessing script — 97e38d0 24 days ago — 221 commits

Dashboard_Data	update schuyler county id	last month
R	update pointer to new file in S3 bucket	9 months ago
oldversions	move master db copy	2 years ago
rsconnect/documents	data and new dash update	14 months ago
www	image for info page	3 months ago
.DS_Store	update	2 years ago
.RData	first commit	2 years ago
.Rhistory	data and new dash update	14 months ago
Master database.csv	Add files via upload	10 months ago
New York State sewersheds with sara...	simplify sewershed geometry	7 months ago
New York State sewersheds with sara...	simplify sewershed geometry	7 months ago
New York State sewersheds with sara...	simplify sewershed geometry	7 months ago
New York State sewersheds with sara...	simplify sewershed geometry	7 months ago
README.md	Initial commit	2 years ago
SARS2EWSP.Rproj	first commit	2 years ago
SARS_dashboard_ww_preprocessing.R	dash preprocessing script	24 days ago
nys.wastewater.new.rds	update test file	7 months ago
test_app.R	5.2.0	last month

Figure 1: Git Hub main layout. The R folder contains the check for updates script.

```r
all.cases$Date <- mdy(all.cases$test_DATE)
all.cases$Cases <- as.numeric(gsub(",", "", all.cases$New.Positives))


# add test positivity
all.cases$tests <- as.numeric(gsub(",", "", all.cases$Total.Number.of.Tests.Performed))
all.cases$Test_positivity <- all.cases$Cases/all.cases$tests

# add active cases
all.cases <- all.cases %>%
    group_by(County) %>%
    mutate(active.cases = purrr::map_dbl(Date, ~sum(Cases[between(Date, . - 6, .)])))

all.cases <- all.cases %>%
    group_by(County) %>%
    mutate(avg_active.cases = purrr::map_dbl(Date, ~mean(active.cases[between(Date,
        . - 7, .)], na.rm = TRUE)))  # 7 day average of active cases

# change st lawrence spelling
all.cases$County[all.cases$County == "St. Lawrence"] <- "St Lawrence"
```

3.4 Spatial data

Spatial data including the sewersheds, NY county borders, and wastewater treatment plant point locations are loaded into the global environment. Copies of each file are stored in the Git Hub repository folder called "data" (see Figure 1).

```r
##### ##### ##### ##### ##### Spatial data load ##### ##### ##### #####

# sewershed shapefile load
catchment <- st_read("Dashboard_Data/New York State sewersheds with saratoga.shp")

# change binghamton johnson city to influent for the dash to work
catchment$Method[catchment$SW_ID == "36007NY0024414C002"] <- "Influent"
catchment$Method[catchment$SW_ID == "36007NY0024414C003"] <- "Influent"

# identifying information for sewersheds (flat file of meta data)
sewer.id <- read.csv("Dashboard_Data/Sewer.IDs.csv", stringsAsFactors = FALSE)

# change binghamton johnson city for sewersheds
sewer.id$Method[sewer.id$SW_ID == "36007NY0024414C002"] <- "Influent"
sewer.id$Method[sewer.id$SW_ID == "36007NY0024414C003"] <- "Influent"

# load wwtp locations
wwtps <- read.csv("Dashboard_Data/NY_WWTP_DEC_List.csv", stringsAsFactors = FALSE)
wwtps <- wwtps %>%
    filter(Plant.Type == "Municipal")
wwtps <- wwtps %>%
    filter(SPDES.Perm %in% sewer.id$WWTP_ID)
wwtps$WWTP_ID <- wwtps$SPDES.Perm

# jitter the points
```

```
wwtps$Latitude <- jitter(wwtps$Latitude, factor = 1)
wwtps$Longitude <- jitter(wwtps$Longitude, factor = 1)

# ny county boundary shapefile
ny_counties <- st_read("Dashboard_Data/Counties_Shoreline.shp")
```

4 User Interface

Shiny has two primary functions: the user interface or UI and the server. The UI is where all the buttons, plots, and other features that users interact with are placed and the server is where each of these items is generated and programmed. For more information on the basics of shiny and other resources, please see the References and Resources section for helpful links.

The shiny dashboard package comes standard with built-in features for a title bar, sidebar, and main body layout. Figure 2 shows the main layout for the NY SARS-2 dashboard.

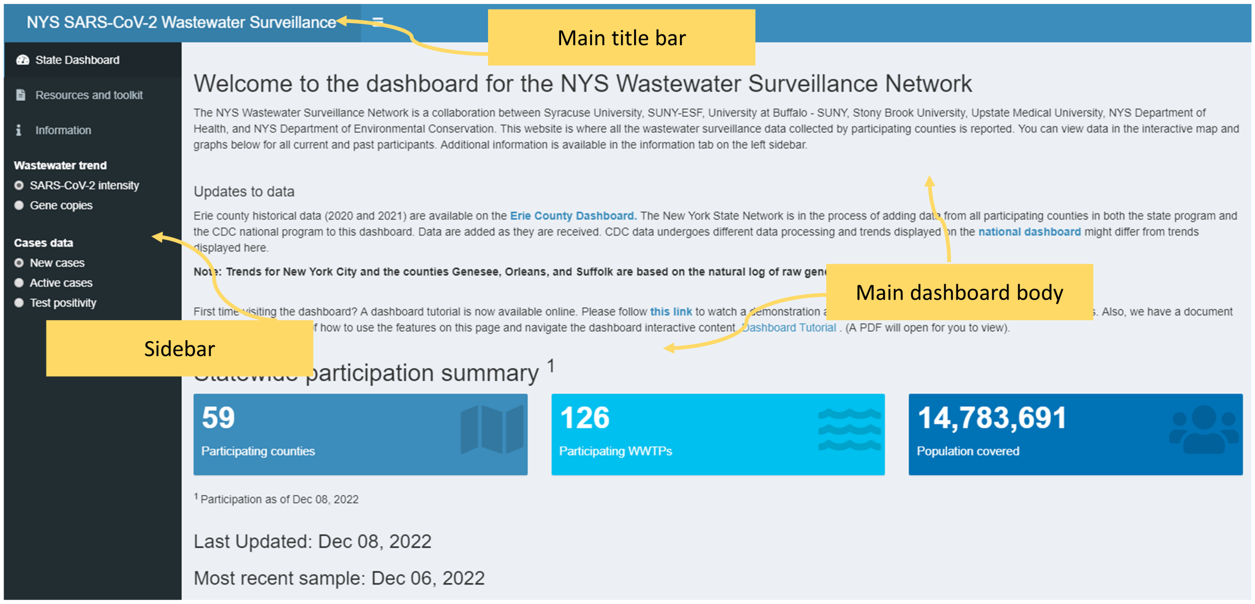

Figure 2: Main dashboard page created by shinydashboard.

Generating the main dashboard page can be created using the code chunk below. Note that the content contains placeholders for you to fill in whatever is relevant for your dashboard. All of the explanatory text and supporting material must be added in the UI usually with HTML code.

```
# Define UI
ui <-
  function(req){
    dashboardPage(
      dashboardHeader(
        title = "Title", # title in the box at top of the page
                      titleWidth = 450), # this titlewidth is linked to the sidebar
```

```
    # SIDEBAR CONTENT #
    dashboardSidebar( width = 225,
                      # SIDEBAR MENU #
                      sidebarMenu(id = "sidebarid",
                                  # STYLE ARGUMENT USES CSS CODE
                                  style = "position:fixed; width:auto; overflow-x: clip;",
                                  # keeps the sidebar content from scrolling
                                  # MENU ITEMS THAT ARE TABS ON THE SIDEBAR
                                  menuItem("State Dashboard", tabName = "dashboard",
                                           icon = icon("dashboard")),
                                  menuItem("Information", icon = icon("info"),
                                           tabName = "Information"),
                                  ### CONDITIONAL PANEL FOR SIDEBAR BUTTONS ON MAINPAGE ###
                                  conditionalPanel(
                                    condition = 'input.sidebarid == "dashboard"'
                                  ) # close conditional panel
                      ) # close sidebar menu
    ), # close dashboard sidebar argument
    dashboardBody(
      # specify new color theme
      use_theme(mytheme),
      useShinyjs(), #for shinyjs code to work
      useShinyalert(),  # Set up shinyalert
      # CSS style arguments (e.g., font size)
      # increase size of acutal map display based on window
      tags$style(type = "text/css", "#NYBetaMap {height: calc(100vh - 80px) !important;}"),
      tags$head(tags$style('.selectize-dropdown {z-index: 10000}')),
      tabItems(
        tabItem(tabName = "dashboard",
                fluidRow(
                  column(12,
                         p("Optional introduction text"),
                  )# end column argument
                ), # end fluid row opening paragraph
                #  VALUE BOXES WITH STATE SUMMARIES  #
                fluidRow(column(width = 12, h2(HTML(paste("Header", "<sup>", "1", "</sup>"))))),
                fluidRow(
                  # number of counties actively reporting
                  valueBox( value = "value_here",
                            subtitle = "Participating counties",
                            color = "light-blue",
                            icon = icon("map")
                  ),
                  # number of  treatment plants reporting
                  valueBox( value = "value_here",
                            #subtitle = "Participating WWTPs",
                            subtitle = "Participating WWTPs",
                            color = "aqua",
                            icon = icon("water")
                  ),
                  # population covered
                  valueBox( value = formatC(1000000),
                            subtitle = "Population covered",
```

```
                              color = "blue",
                              icon = icon("users"))
                  ), # end fluid row summary boxes
          ), # end tab item
          # another tab page
          tabItem(tabName = "resources",
                  fluidRow(
                    column(12,
                           h3("Wastewater surveillance toolkit"),
                           br(),
                           p("Maybe we can provide resources here?")
                    )
                  )
          ),
        )# end tab items plural
      ) # end dashboard body
    )# end dashboardPage
  } # close function for ui
```

4.1 Customizing Shiny Dashboard colors

The shiny dashboard package provides a default color scheme, but you can modify this using the package fresh. This package lets you modify the colors for the sidebar, title panel, and other features.

```
# define new dashboard theme
library(fresh)

# NEW DASHBOARD COLOR THEME #
# We can specify the colors here for the dashboard content theme
mytheme <- create_theme(
  adminlte_color(
    light_blue = "#698b69" # title bar color
  ),
  adminlte_sidebar(
    width = "225px",
    dark_bg = "#68838b", # sidebar background color
    dark_hover_bg = "#81A1C1", # selected page button color and hover button color
    dark_color = "#2E3440" # unselected button text color,
  ),
  adminlte_global(
    content_bg = "#EEEE0", # main background color of dashboard body
    box_bg = "#f0ffff", # dashboard boxes color
    info_box_bg = "#D8DEE9" # info boxes color
  )
)

# call the theme in the dashboard body function in the UI
 dashboardBody(
        # specify new color theme
        use_theme(mytheme)
 )
```

4.2 Value boxes

Shiny dashboard allows developers many different ways to customize the look of their dashboard. One feature that is particularly nice for providing quick summaries are value boxes. These can contain static values showing a number with an associated icon or reactive values that are generated in the global environment or the server. Icons that are compatible with Shiny dashboard value boxes can be found from Glyphicon or Font Awesome.

```
valueBox(value = value_object, subtitle = "subtitle", color = "light-blue", icon = icon("map"))
```

4.3 Drop-down menu (select input)

Shiny contains many interactive features. One that we use in our dashboard is “select input” that allows users to select an option from a drop-down to inititiate some reaction. In the SARS-2 dashboard, users can change the colors and legend on the map between two different options.

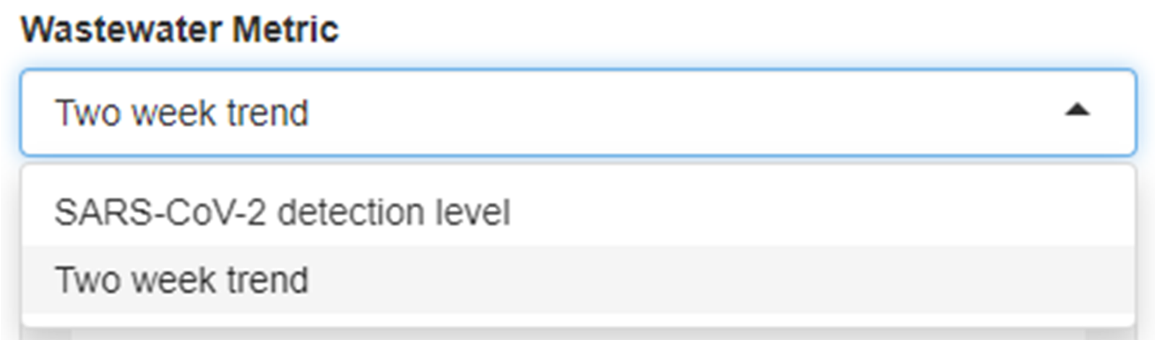

Figure 3: Select input options in the SARS-2 dashboard.

Like other Shiny content, the drop-down is created in the UI and the interactive output is programmed in the server portion of the code.

```
ui <- function(req) {
    selectInput(inputId = "state_map_toggle", label = "Wastewater Metric", choices = c(`SARS-CoV-2 dete
        `Two week trend` = "Trend_factor"), selected = "SARS-CoV-2 detection level")
}

server <- function(input, output, session) {

    # color palettes and levels for categorical variables create color level
    # and value vectors for each factor variable
    trend_colors <- c("blue", "dodgerblue3", "cornflowerblue", "lightskyblue", "papayawhip",
        "khaki2", "orange", "orangered", "orangered4")
    trend_levels <- c("< -75%", "-50% to -75%", "-25% to -50%", "-10% to -25%", "-10% to 10%",
        " 10% to 25%", " 25% to 50%", " 50% to 75%", "> 75%")
    alert_colors <- c("plum3", "darkorange2", "red")
    alert_levels <- c("Low", "Moderate", "Substantial to High")
```

```r
    # change the color palette according to the select input choices
    if (input$state_map_toggle == "Alert_factor") {
        wastewater_pal <- colorFactor(alert_colors, levels = alert_levels, ordered = TRUE)
    } else if (input$state_map_toggle == "Trend_factor") {
        wastewater_pal <- colorFactor(trend_colors, levels = trend_levels, ordered = TRUE)
    }
}


# Run the application
shinyApp(ui = ui, server = server)
```

5 Leaflet

The NY SARS-2 dashboard has most of its functionality linked to the Leaflet map where users can focus on geographies or communities of interest to them. For example, users can click on sampling locations that represent a community of interest, or a county of interest to see trends and recent results for SARS-CoV-2 levels. Interaction with the Leaflet map displays information in popups and then when users scroll down the page (Figure 3), they can see trend graphs that are linked to the location that was selected.

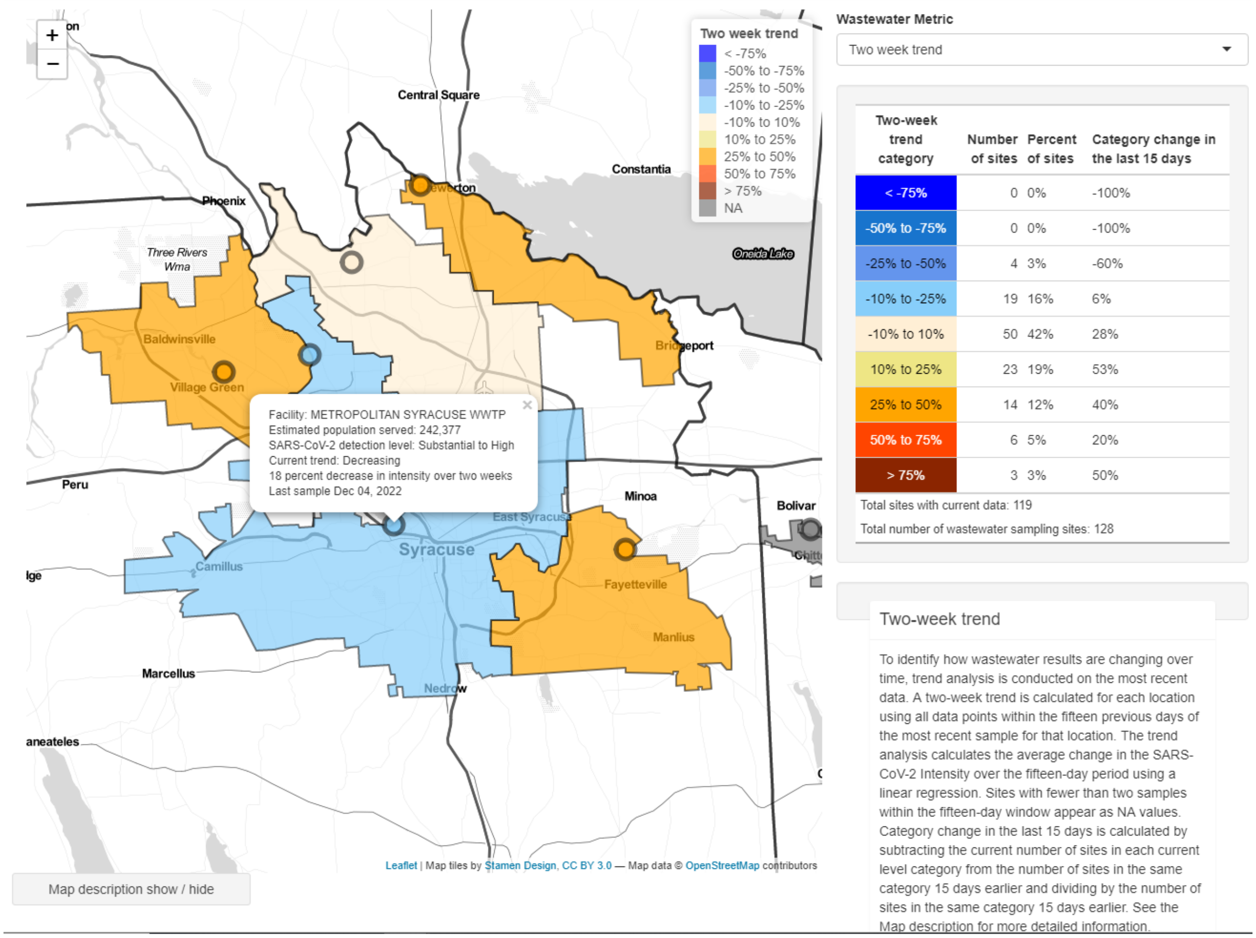

Figure 4: Leaflet map with example popup text.

The Leaflet map is created with the following code chunk:

```
###### LEAFLET MAP OF NEW YORK #######

 # create map for renders
 nybetamap_preset <- leaflet(data = ny_counties, options = leafletOptions(
   zoomControl = TRUE, maxZoom = 13, minZoom = 6,
   dragging = TRUE)
   ) %>%
   addMapPane("nymap", zIndex = 420) %>%
   addMapPane("tiles", zIndex = 400) %>%
   addMapPane("catchment", zIndex = 422) %>%
   addMapPane("wwtps", zIndex = 435) %>%
   addPolygons(data = ny_counties,
               smoothFactor = 0.2, fillOpacity = 0.7,
               fillColor = ~fact_pal(Participating),
               stroke = TRUE,
               color = "black",
               weight = 1,
               layerId = ny_counties$County,
               options = pathOptions(pane = "nymap", clickable = TRUE),
               label = ny_counties$County,
               group = "County status",
               highlightOptions = highlightOptions(color = "white", weight = 2,
                                                    bringToFront = TRUE)) %>%
   addLegend(position = c("topright"), pal = fact_pal,
             values = ~Participating, #
             opacity = 0.7, #na.label = "NA",
             title = "Surveillance status",
             group = "County status",
             layerId = "legend_participating") %>%
   addProviderTiles(providers$Stamen.TonerLite, group = "Base map",
                    options = c(pathOptions(pane = "tiles"))) %>%
   addProviderTiles(providers$Stamen.TonerLines, group = "Base map",
                    options = c(providerTileOptions(opacity = 0.35),
                                pathOptions(pane = "tiles")))%>%
   addProviderTiles(providers$Stamen.TonerLabels,group = "Base map",
                    options = pathOptions(pane = "tiles")) %>%
   # counties appear through zoom levels 3 to 10 then become transparent
   groupOptions("County status", zoomLevels = 3:10) %>%
   # add counties to appear transparent at lower zoom
   addMapPane("county_transparent", zIndex = 421) %>%
   addPolygons(data = ny_counties,
               smoothFactor = 0.2, #fillOpacity = 0.7,
               fillColor = "transparent",
               stroke = TRUE,
               color = "black",
               weight = 3,
               layerId = ny_counties$FIPS_CODE,
               group = "County transparent",
               options = pathOptions(pane = "county_transparent",
                                     clickable = FALSE) # cannot click counties
   ) %>%
   # transparent counties appear at zoom levels 11 to 15
```

```
    groupOptions("County transparent", zoomLevels = 11:15)
```

5.1 Leaflet interactivity

The leaflet map can be manipulated using the mouse to "zoom" in to see smaller geographies. In addition, polygons and markers can be clicked to display more information.

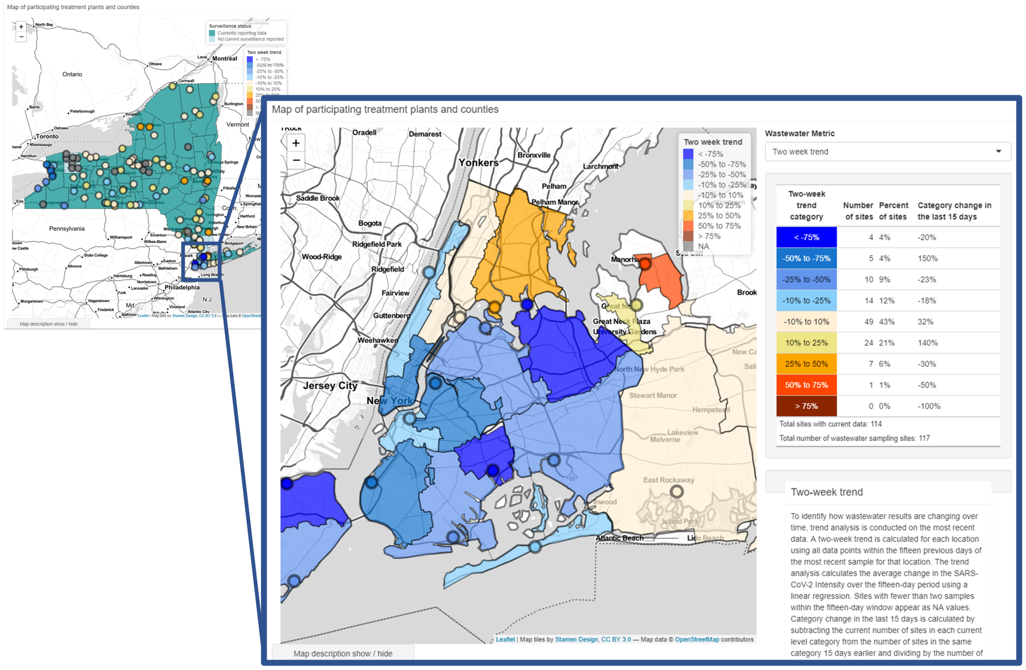

Figure 5: Zooming in reveals more information about sampling locations including sewershed boundaries showing what communities are covered by surveillance.

5.2 Leaflet proxy

Layers on the Leaflet map for the wastewater treatment plants and sewersheds change color with the drop-down menu. This updates the map using Leaflet proxy.

```
  # palette for county participation
  fact_pal <- colorFactor(c("cyan4", "light blue", "black"),
                          levels = c("Currently reporting data",
                                     "No current surveillance reported",
                                     "Not currently participating"))

  #### SHOW/HIDE ALERT LEVELS IF SELECT INPUT IS ON COMMUNITY TRANSMISSION ####
  observe({
    ##### TWO COLOR FACTOR SETS BASED ON USER INPUT FOR WHAT PATTERN TO DISPLAY ####
    if (input$state_map_toggle == "Alert_factor") {
      wastewater_pal <- colorFactor(alert_colors,
                                    levels = alert_levels, ordered = TRUE)
    } else if (input$state_map_toggle == "Trend_factor") {
```

```
    wastewater_pal <- colorFactor(trend_colors,
                                  levels = trend_levels,
                                  ordered = TRUE)    }
  proxy_map <- leafletProxy("NYBetaMap") %>%
    # add new markers with the trend level
    addCircleMarkers(data = wwtps_active ,  ~Longitude, ~Latitude,
                     fillColor =~wastewater_pal(eval(as.symbol(input$state_map_toggle))),
                     fillOpacity = 0.9,
                     color = "black", #
                     opacity = 0.5,
                     popup = paste("Facility:", (as.character(wwtps_active$Facility.N)), "<br>",
                                   "Estimated population served:",
                                   formatC(wwtps_active$pop_served_2, format = "d",
                                           big.mark = ","),
                                   "<br>",
                                   "SARS-CoV-2 detection level: ",
                                   wwtps_active$Alert_factor, "<br>",
                                   "Current  trend:",
                                   ifelse(wwtps_active$TwoWeekBet > 0, "Increasing", "Decreasing"),
                                   "<br>",
                                   round(abs((wwtps_active$TwoWeekBet) ) * 100, 0), "percent",
                                   ifelse(wwtps_active$TwoWeekBet > 0,
                                          "increase", "decrease"), "in ",
                                   ifelse(wwtps_active$trend_measure == "raw gene copies",
                                   "natural logof raw gene copies",
                                          "intensity")  ," over two weeks", "<br>",
                                   "Last sample",
                                   lubridate::as_date(wwtps_active$Date_collected) %>%
                                   format('%b %d, %Y')
                                   ),
                     label = ~paste(as.character(Facility.N)),
                     layerId = wwtps_active$WWTP_ID,
                     options = pathOptions(pane = "wwtps"),
                     group = "WWTPs"
    )  %>%
    groupOptions("Catchments", zoomLevels = 11:15) %>%
    addLegend(data = wwtps_active, position = c("topright"),
              pal = wastewater_pal,
              values = ~eval(as.symbol(input$state_map_toggle)),
              opacity = 0.7,
              title = ifelse(input$state_map_toggle == "Alert_factor",
                             "SARS-CoV-2 detection level",
                             "Two week trend"),
              group = "WWTP",
              layerId = "legend_wastewater"
    )
})
```

6 Trend graphs and tables

Most wastewater results that are relevant to users are presented in the Leaflet map, however trends and historical data can help interpret recent results. Trends are presented in two plots (raw gene copies and

fecal-normalized wastewater intensity) in the graphs below the Leaflet map. These can be switched using a toggle on the left of the dashboard in the sidebar.

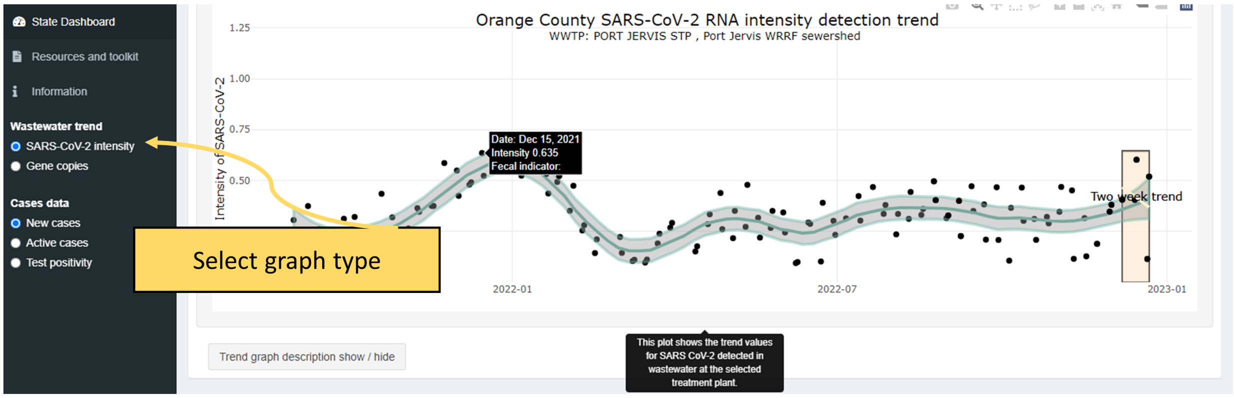

Figure 6: Trend graph and selection buttons.

6.1 Wastewater trends

Each graph is generated by a function that runs each time a location is clicked on the Leaflet map. Clicked location IDs are then used to filter dataframes to only have data for the clicked location and these dataframes are used to generate ggplots that are made interactive using Plotly.

```
###### WW PLOT OF INTENSITY FOR RECENT DATA #####
  sewershed_ww_plot_function_log_recent <- function(plot_dataframe){
    sewershed_sars_plot <- ggplot(plot_dataframe, aes (x = lubridate::as_date(Date),
                                                       y = IntensityDNA, group = 1,
                                                       text = paste0(
                                                         "Date: ", lubridate::as_date(Date) %>%
                                                           format('%b %d, %Y'), "<br>",
                                                           "Intensity ", round(IntensityDNA, 3),
                                                         "<br>",
                                                         "Fecal indicator: ", lab_method
                                                       )# end paste0 call
    )# end aes call
    ) +
      geom_rect(data = plot_dataframe %>%
                  filter(lubridate::as_date(Date) %in%
                           lubridate::as_date(wastewater.df.sewersheds$Date)),
                aes(fill = factor(Trend_factor),
                    ymin = 0, ymax = max(plot_dataframe$IntensityDNA),
                    xmin = min(plot_dataframe$maxdate) - days(15),
                    xmax = max(plot_dataframe$maxdate)
                    ),
                color = "black",
                alpha = 0.1)+
      # points
      geom_point(color="black") +
      # smoothed line
      geom_smooth(color="#69b3a2", se = TRUE,
                  span = ifelse(
```

```r
                  length(plot_dataframe$Date) >4 & length(plot_dataframe$Date) <= 25, 0.75,
                                                        0.25),
                method = ifelse(length(plot_dataframe$Date) <=4, "lm", "loess")) +
  geom_point(data = plot_dataframe %>%
                 filter(lubridate::as_date(Date) %in%
                        lubridate::as_date(wastewater.df.sewersheds$Date)),
               aes(x = lubridate::as_date(Date),
                   y = IntensityDNA), color = "black")+
  ggplot2::geom_smooth(data = plot_dataframe %>%
                       filter(Date >= maxdate-days(15)),
                     aes(x = lubridate::as_date(Date),
                         y = IntensityDNA, color = factor(Trend_factor)),
                     method = "lm", se = FALSE)+
  scale_color_manual(values = c("< -75%" = "blue", "-50% to -75%" = "dodgerblue3",
                              "-25% to -50%" = "cornflowerblue", "-10% to -25%"
                              = "lightskyblue", "-10% to 10%" = "papayawhip",
                              " 10% to 25%" = "khaki2", " 25% to 50%" = "orange",
                              " 50% to 75%" = "orangered", "> 75%" ="orangered4"))+
  ylab("Intensity of SARS-CoV-2")+
  xlab("")+
  theme_minimal()+
  theme(legend.position = "none")+
  scale_y_continuous(#limits = c(0, NA),
    expand = expansion(mult = c( 0, 1 ))) +
  xlim(min(lubridate::as_date(plot_dataframe$Date)),
       max(lubridate::today(tzone = 'America/New_York') %>% format('%b %d, %Y'))
       ) +
  scale_fill_manual(values = c("< -75%" = "blue", "-50% to -75%" = "dodgerblue3",
                             "-25% to -50%" = "cornflowerblue", "-10% to -25%"
                             = "lightskyblue", "-10% to 10%" = "papayawhip",
                             " 10% to 25%" = "khaki2", " 25% to 50%" = "orange",
                             " 50% to 75%" = "orangered", ">75%"="orangered4") ) +
  annotate(geom = "text",
           label = "Two week trend",
           x = min(plot_dataframe$maxdate - days(7)),
           y = median(plot_dataframe$IntensityDNA) + 0.1,
           expand = c(1,1))

# change to plotly
sewershed_plotly_default <- ggplotly(sewershed_sars_plot,
                                     tooltip = c("text")) %>%
  layout(title = list(text = paste0(paste("<br>",plot_dataframe$County,
                      "County", "SARS-CoV-2 RNA intensity detection trend",sep = " "),
                       '<br>',
                       '<sup>',
                       paste("WWTP:", plot_dataframe$WWTP, ",",
                       ifelse(length(unique(plot_dataframe$Sewershed))==1,
                              plot_dataframe$Sewershed, "" ),
                       "sewershed",  sep = " "),'</sup>','<br>'
  )
  )
  )
sewershed_plotly_default <- sewershed_plotly_default %>%
```

```
    style(hoverinfo = "skip", traces = 1) %>%
    style(hoverinfo = "skip", traces = 2) %>%
    style(hoverinfo = "skip", traces = 5) %>%
    style(hoverinfo = "skip", traces = 6)%>%
    style(hoverinfo = "skip", traces = 8)

  return(sewershed_plotly_default)
}

default_ww_plot_log <-sewershed_ww_plot_function_log_recent(sewershed_plot)
output$sewershed_plotly_default_log <- renderPlotly(default_ww_plot_log)
```

6.2 Case trends

Case data graphs are generated using a similar function.

```
# new cases
case_plot_function <- function(case_dataframe) {
    case_county_plot <- ggplot(case_dataframe, aes(x = lubridate::as_date(Date),
        y = Cases, group = 1, text = paste0("Date: ", lubridate::as_date(Date) %>%
            format("%b %d, %Y"), "<br>", "New cases ", Cases)  # end paste0 call
)) +
        geom_bar(position = "dodge", stat = "identity") + theme(plot.background = element_blank()) +
        theme(panel.background = element_blank()) + theme(panel.grid.major.y = element_line(color = "gr
        xlab("") + ylab("New cases (total)") + labs(caption = "") + theme(axis.text.x = element_text(hj
        text = element_text(size = 12)) + theme_minimal() + xlim(min(lubridate::as_date(case_dataframe$
        max(lubridate::today(tzone = "America/New_York") %>%
            format("%b %d, %Y")))

    case_county_plot

    case_plotly_default <- ggplotly(case_county_plot, tooltip = c("text")) %>%
        layout(title = list(text = paste0(paste("<br>", case_dataframe$County, "County reported new cas
            sep = " "), "<br>", "</sup>", "<br>")))
    return(case_plotly_default)
}
```

6.3 Summary tables

In addition to maps and graphs, we also include several tables in the dashboard for users to see summary information. These tables are created with the "gt" package that uses html code to produce output that can be customized.

These tables can be created and stored as images that the Shiny app will render.

```
ui <- function(req) {
    gt_output("alert_table")  # generate the table in the UI
}

server <- function(input, output, session) {

    # create and store table
```

Wastewater Metric

SARS-CoV-2 detection level

Detection level category	Number of sites	Percent of sites	Category change in the last 15 days
No data	0	0%	NA
Low	0	0%	NA
Moderate	7	6%	133%
Substantial to High	119	94%	-3%

Total sites with current data: 126

Total number of wastewater sampling sites: 128

Figure 7: Example table showing alert level summary information.

```
    table_alert <- percent_change_transmission %>%
        gt() %>%
        data_color(columns = "Detection level category", colors = scales::col_factor(alert_colors,
            levels = alert_levels, ordered = TRUE)) %>%
        tab_source_note(paste("Total sites with current data:", sum(percent_change_transmission$`Number
        tab_source_note(paste("Total number of wastewater sampling sites:", length(unique(ww_transmissi
        tab_style(style = cell_text(weight = "bold"), locations = list(cells_column_labels(gt::everythi
        # change font size
    tab_options(table.font.size = 14)

    # render table
    output$alert_table <- render_gt(table_alert)
}

# Run the application
shinyApp(ui = ui, server = server)
```

7 Interactivity between Leaflet and plots

Clicking the markers on the Leaflet map will update the trend graphs lower down on the dashboard page.

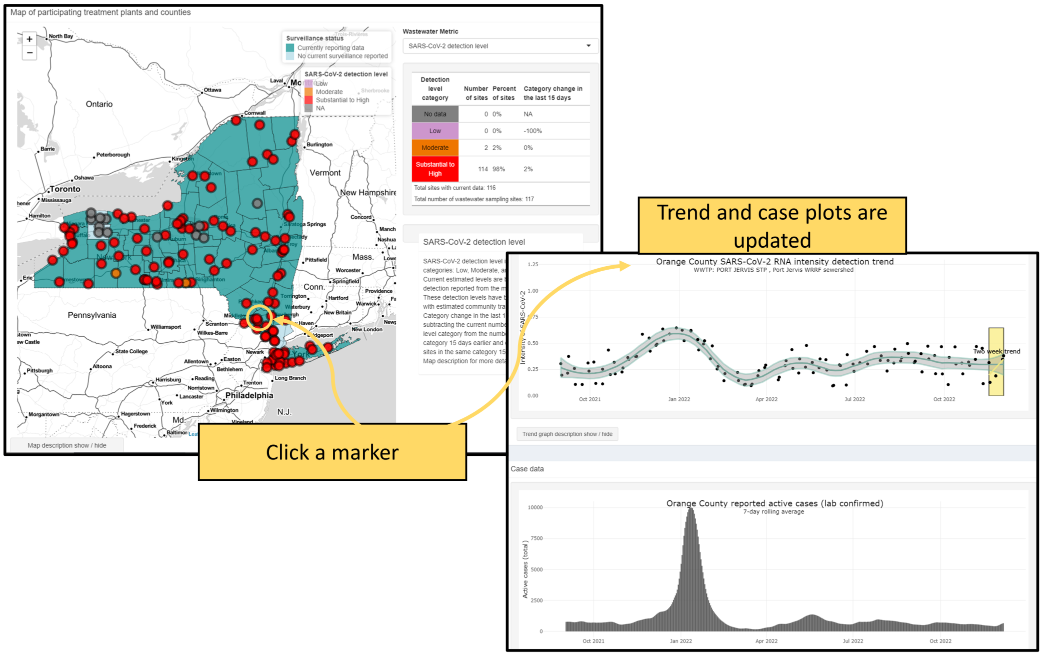

Figure 8: Leaflet and trend graph interaction.

Clicking on the Leaflet map is linked to other Shiny operations including updating the trend graphs to show data for the selected location. Each layer in the leaflet map has an ID “layerid”) that can be called using the function mapname_shape_click or mapname_marker_click. This will capture the ID of the layer and that can be used to filter dataframes or other functions.

```
observeEvent(input$NYBetaMap_shape_click, {
    # capture the info of the clicked polygon
    click <- input$NYBetaMap_shape_click
    sewershed_plot <- wastewater.historic %>%
        filter(County == click$id)
    # stop if empty df
    req(!is.na(sewershed_plot$trend_value))
    # grab county data
    county.case <- all.cases %>%
        filter(County %in% sewershed_plot$County) %>%
        filter(Date >= min(sewershed_plot$Date))
    case_plotly <- case_plot_function(case_dataframe = county.case)
    active_plotly <- case_active_plot_function(case_dataframe = county.case)
    positivity_plotly <- case_positivity_plot_function(case_dataframe = county.case)
    # return case plotly
    if (!is.null(click$id)) {
        output$county_case_plotly <- renderPlotly(case_plotly)
    }
    # return active plotly
    if (!is.null(click$id)) {
        output$county_active_plotly <- renderPlotly(active_plotly)
    }
    # return positivity plotly
    if (!is.null(click$id)) {
        output$county_positivity_plotly <- renderPlotly(positivity_plotly)
    }
})
```

8 Closing thoughts

This tutorial provides the basic information on how the NY SARS-2 dashboard was designed using R Shiny and Leaflet for R. This tutorial is not exhaustive nor is it meant to replace existing courses or learning materials. It is meant to explain and outline the choices we made when developing our dashboard. Elements, function, and design choices can be adapted by readers for their own dashboards. Further, we recommend the reader review the reference and resource list. There are several helpful package documentations cited including for R Shiny Dashboard and NY SARS-2 Dashboard. We hope this tutorial will help others in their efforts to build and deploy dashboards for public health and other purposes. Full dashboard code is available at the following webpage: _____ and the live dashboard can be reached by visiting: NY SARS-2 Dashboard.

9 References

Attali, D. (2021). shinyjs: Easily Improve the User Experience of Your Shiny Apps in Seconds (2.1.0).https://CRAN.R-project.org/package=shinyjs

Attali, D., & Edwards, T. (2021). shinyalert: Easily Create Pretty Popup Messages (Modals) in “shiny” (3.0.0) [R].https://CRAN.R-project.org/package=shinyalert

Bache, S. M., & Wickham, H. (2022). magrittr: A Forward-Pipe Operator for R (2.0.3) [R].https://CRAN.R-project.org/package=magrittr

Bailey, E. (2022). ShinyBS: Twitter Bootstrap Components for shiny (0.61.1) [R].https://CRAN.R-project.org/package=shinyBS

Chang, W., & Borges Ribeiro, B. (2021). shinydashboard: Create Dashboards with “shiny” (0.7.2) [R].

Chang, W., Cheng, J., Allaire, J. J., Sievert, C., Schloerke, B., Xie, Y., Allen, J., McPherson, J., DiPert, A., & Borges, B. (2021). shiny: Web Application Framework for R (1.7,1).

Cheng, J., Karambelkar, B., & Xie, Y. (2022). leaflet: Create Interactive Web Maps with the JavaScript 'Leaflet" Library (2.1.1) [R].https://CRAN.R-project.org/package=leaflet

Cheng, J., Sievert, C., Schloerke, B., Chang, W., Xie, Y., & Allen, J. (2021). htmltools: Tools for HTML (0.5.2).https://CRAN.R-project.org/package=htmltools

DOH. (2022). COVID-19 Data in New York | Department of Health. https://coronavirus.health.ny.gov/covid-19-data-new-york Granjon, D. (2021). shinydashboardPlus: Add More “AdminLTE2” Components to “shinydashboard” (2.0.3) [R]. https://CRAN.R-project.org/package=shinydashboardPlus

Grolemund, G., & Wickham, H. (2011). Dates and Times Made Easy with lubridate. Journal of Statistical Software, 40, 1–25. https://doi.org/10.18637/jss.v040.i03

Henry, L., & Wickham, H. (2020). purrr: Functional Programming Tools (0.3.4) [R]. https://CRAN.R-project.org/package=purrr Iannone, R., Cheng, J., & Schloerke, B. (2022). gt: Easilty Create Presentation-Ready Display Tables (0.6.0) [R]. https://CRAN.R-project.org/package=gt

Karambelkar, B., & Schloerke, B. (2018). leaflet.extras: Extra Functionality for “leaflet” Package (1.0.0) [R]. https://CRAN.R-project.org/package=leaflet.extras

Larsen, D. A., Collins, M. B., Du, Q., Hill, D., Insaf, T. Z., Kilaru, P., Kmush, B. L., Middleton, F., Stamm, A., Wilder, M. L., Zeng, T., & Green, H. (2022). Coupling freedom from disease principles and early warning from wastewater surveillance to improve health security. PNAS Nexus, 1(1), pgac001. https://doi.org/10.1093/pnasnexus/pgac001

Leeper, T. J. (2020). aws.s3: AWS S3 Client Package (0.3.21) [R].

Pebesma, E. (2018). Simple features for R: Standardized support for spatial vector data. The R Journal, 10(1), 439–446. https://doi.org/10.32614/RJ-2018-009

Sail, A., & Attali, D. (2020). shinycssloaders: Add Loading Animations to a “shiny” Output while It's Recalculating (1.0.0) [R]. https://CRAN.R-project.org/package=shinycssloaders

Sievert, C. (2020). Interactive Web-Based Data Visualization with R, plotly, and shiny. Chapman and Hall/CRC. https://plotly-r.com

Wickham, H. (n.d.). ggplot2: Elegant Graphics for Data Analysis. Springer-Verlag New York. https://ggplot2.tidyverse.org

Wickham, H. (2019). stringr: Simple, Consistent Wrappers for Common String Operations (1.4.0) [R]. https://CRAN.R-project.org/package=stringr

Wickham, H., Francois, R., Henry, L., & Muller, K. (2022). dplyr: A Grammar of Data Manipulation (1.0.9) [R]. https://CRAN.R-project.org/package=dplyr

Wickham, H., & Girlich, M. (2022). tidyr: Tidy Messy Data (1.2.0) [R]. https://CRAN.R-project.org/package=tidyr
