# Supplementary material for: Operationalizing an open-source dashboard for communicating results of wastewater-based surveillance
Source: MethodsX. 2023 Jul 27;11:102299. doi: 10.1016/j.mex.2023.102299 (PMC10404718; doi:10.1016/j.mex.2023.102299)
Supplement: Supplementary file 2 [file mmc2.docx]

| Table 2 – Estimated costs for development and maintenance of R shiny dashboard. | | |
| --- | --- | --- |
| PHASE 1 - Initial prototype development | | |
| Item | **Cost** | **Details** |
| Personnel time* | $6,000.00 | 150 person hours at $40 per hour |
| AWS bucket | $2.00 | Very low cost, <$1 per month |
| R shiny tier – free | - | Free tier used for development |
| Total | $6,002.00 | Approximately two months of time / 150 person hours |
|  |  |  |
| PHASE 2 - Final version development |  |  |
| Item | Cost | Details |
| Personnel time | $12,000.00 | 300 person hours at approximately $40 per hour |
| AWS Bucket | $3.00 | Very low cost, <$1 per month |
| R shiny tier – basic | $147 | Expanded with additional in-house users added. ($49 per month) |
| Total | $12,150.00 | Approximately three months / 300 person hours |
|  |  |  |
| PHASE 3 - Ongoing maintenance after development and public launch | | |
| Personnel time – Maintenance, data management, additional web development | $2,720.00 | Maintenance of existing data and web infrastructure, response to queries, bug reports, manage new site additions, new data additions, etc.  Approximately 68 person hours per month at approximately $40 per hour |
| AWS bucket | $2.00 | Very low cost, <$2 per month |
| Custom domain name (optional) | $1.67.00 | Optional addition. Not needed for development phase. Approximately $20.00 per year |
| R Shiny tier – professional | $349 per month | Expanded for live deployment to serve public visits |
| Total (per month) | $3,072.67 | Approximately $36,872.00 per year. |
| *Notes: *Personnel time is based on a rate of approximately $40 per hour or an annual salary of $83,200 for one person. For our project, two developers and one supervisor participated. The developers were compensated at approximately this rate as part of their salary along with additional work. The supervisor participated in meetings as per their regular duties. During Phase 1 and Phase 2, Developer 1 spent approximately 50-75% of their time on the development with Developer 2 spending approximately 10-25% of their time on development.*  *During Phase 3, two developers spent at most 25% of their time or 8 hours per week on maintenance, data management, and web development. This includes data management related to the overall activity and integrity of the wastewater data for New York State beyond SARS-CoV-2.* | | |
